# Encephalitic Alphaviruses: Epidemiology, Pathogenesis and Vaccine Development

**DOI:** 10.3390/vaccines14070580

**Published:** 2026-06-30

**Authors:** Nouha Kisra, Zoe de Zeeuw, George Eustace, Rose Gladman, Yong Ji, Sthefany Pagliari, Young Chan Kim

**Affiliations:** 1 St Hilda’s College, University of Oxford, Cowley Place, Oxford OX4 1DY, UK; nouha.kisra@st-hildas.ox.ac.uk; 2 Hertford College, University of Oxford, Cattle Street, Oxford OX1 3BW, UK; zoe.dezeeuw@hertford.ox.ac.uk; 3 Magdalen College, University of Oxford, High Street, Oxford OX1 4AU, UK; george.eustace@outlook.com; 4 Lincoln College, University of Oxford, Turl Street, Oxford OX1 3DR, UK; rose.gladman@lincoln.ox.ac.uk; 5 Oxford Vaccine Group, Department of Paediatrics, University of Oxford, Oxford OX3 7LE, UK; yong.ji@paediatrics.ox.ac.uk (Y.J.); sthefany.pagliari@paediatrics.ox.ac.uk (S.P.); 6 Centre for Human Genetics, Division of Structural Biology, University of Oxford, Roosevelt Drive, Oxford OX3 7BN, UK

**Keywords:** encephalitic alphaviruses, alphavirus, Eastern equine encephalitis virus (EEEV), Western equine encephalitis virus (WEEV), Venezuelan equine encephalitis virus (VEEV), vaccines, public health, diagnosis, arbovirus

## Abstract

Eastern, Venezuelan, and Western equine encephalitis viruses (EEEV, VEEV, and WEEV) are encephalitic alphaviruses transmitted by mosquitoes throughout the Americas. Infection by these viruses can present in humans as a febrile illness; however, it may progress into potentially life-threatening encephalitis. Currently, no publicly licensed vaccines are available, and at-risk individuals are restricted to superseded vaccines. Here, we will review recent advances in our understanding of how these viruses spread among animal populations and cause disease, and how we can manage their diagnosis and treatment. Additionally, we have summarised the recent developments in vaccines against these viruses in both pre-clinical and clinical stages. Overall, global climate change and ecological disruption drive a need for public access to safe and effective vaccines against EEEV, VEEV, and WEEV, which novel platforms, such as mRNA and viral vectors, may be able to achieve.

## 1. Introduction

Encephalitic alphaviruses are composed of Eastern equine encephalitis virus (EEEV), Venezuelan equine encephalitis virus (VEEV), and Western equine encephalitis virus (WEEV), three neurotropic, enveloped, positive-sense, single-stranded RNA (+ssRNA) viruses all originally identified in the Americas in the 1930s [1,2,3]. Initially mistaken as equine botulism, these viruses were later proved to comprise their own niches, exhibiting serological distinction from one another. This was demonstrated by extensive cross-immunity and cross-immunisation experiments, primarily carried out in horses and guinea pigs, the latter being exceptionally susceptible to the viruses [4].

**Figure 1:**
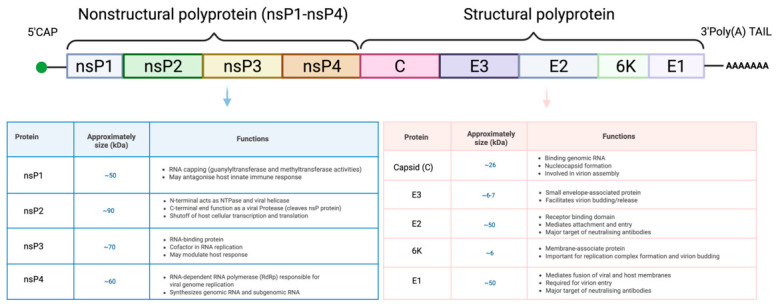
The encephalitic alphavirus genome.

The genomes of encephalitic alphaviruses are arranged into two open reading frames (ORFs), with the 5′ non-structural ORF encoding the viral replication machinery and the 3′ structural ORF encoding the proteins required for virion assembly (Figure 1).

The 5′ ORF is translated directly from the genomic 49S RNA, producing a polyprotein that is subsequently cleaved into four nsPs. Together, these proteins form the viral replication complex, which mediates RNA synthesis and contributes to evasion of host antiviral response. These regions are separated by an internal promoter that drives transcription of the 26S subgenomic RNA. The 5′ ORF is translated directly from the genomic 49S RNA into a polyprotein that is processed into four non-structural proteins (nsP1–nsP4). Together, these proteins form the viral replication complex, with nsP1 functioning in RNA capping and membrane association, nsP2 acting as a protease, helicase, and antagonist of host antiviral signalling, nsP3 facilitating replication complex assembly and host interactions, and nsP4 serving as the RNA-dependent RNA polymerase (RdRP) responsible for viral RNA synthesis. In most alphaviruses, translation of nsP4 is regulated by a leaky stop codon located downstream of nsP3 [5].

Infection begins with viral attachment to host cells, followed by clathrin-mediated endocytosis and release of the viral genome into the cytoplasm. The genomic RNA serves as a template for translation of the nsPs and for synthesis of negative-strand RNA intermediates, which are subsequently used to generate new genomic RNA and abundant 26S subgenomic RNA. The subgenomic RNA, typically produced at higher levels than genomic RNA, functions as the messenger RNA for the structural proteins. Translation of the 3′ ORF yields a structural polyprotein that is cleaved into the capsid (C) protein, envelope glycoproteins E3, E2, and E1, and the small 6K peptide. These proteins mediate virion assembly, encapsidation of newly synthesized genomic RNA, and budding of mature virus particles from the host cell. The efficient expression of structural proteins from the 26S subgenomic RNA is a key feature of alphavirus biology and has been widely exploited in the development of alphavirus-based vaccine vectors [6].

The capsid protein forms the core of the viral capsule. The E3-E2 genes are translated into a single polyprotein that is post-translationally cleaved at a furin site between the E3 and E2 proteins. The E2 and E1 proteins form the spike proteins that are stabilised by the E3 assembly protein, while the 6K protein facilitates the release of the viral progeny [7]. The capsid protein has also been implicated in cytotoxicity and inhibition of host cell transcription, potentially to suppress the interferon response [8]. This suppression may be achieved through capsid interactions with RNA polymerase II (RNAP II) and eukaryotic initiation factor 2 (eIF2), which regulate transcription and translation of interferons and interferon-stimulated genes, respectively. This is supported by capsid-deletions in vaccines resulting in improved immune responses [6]. In particular, the presence of nuclear localisation signals (NLS) in the N-terminal region appears crucial [9,10]. This is supported by the fact that EEEV strains with NLS deletions in their capsids showed delayed replication and increased sensitivity to interferon responses in Vero cells and mice [11].

Variations in structural and nonstructural proteins can cause changes to the pathogenicity and virulence of encephalitic alphaviruses. Johnson et al. demonstrated that single mutations in the E2 protein led to attenuated VEEV virulence in mice [12]. This may be because these mutations disrupt the receptor-binding activity of VEEV, leading to reduced lympho- and neurotropism. Meshram et al. showed that deletions in the hypervariable domains (HVDs) of nsP3 rendered EEEV avirulent in mice [13]. This may be due to changes in EEEV’s ability to recruit host cell proteins, which inhibits its replication in the brain and thus reduces its neurovirulence. Collectively, these facts illustrate the importance of structural and nonstructural proteins in the pathogenesis of encephalitic alphaviruses.

Since their discovery, these viruses have been responsible for a range of sporadic outbreaks of varying size, intensity, and location. EEEV has the highest case fatality rate among humans, with 30–75% of those infected developing lethal encephalitis [14]. EEEV is also the least prevalent, whereas VEEV is by far the most prevalent, predominantly occurring in the form of sporadic outbreaks [15].

The need for effective therapeutics and vaccines against encephalitic alphaviruses has long gone unmet due, in part, to the sporadic nature of the outbreaks and the mitigation of bioterrorism concerns following international agreements. As a result of this, at-risk personnel (such as those in the military or laboratory workers working with these viruses) are still receiving superseded vaccines with low immunogenicity and undesirable reactogenicity, with no vaccines currently licensed for public use. The live-attenuated vaccine used for at-risk personnel against VEEV was first produced by passaging the virus through guinea pig heart cell culture 83 times in 1967 and is still in use today [16,17]. Currently, under the classification of an “Investigational New Drug (IND)”, the 57-year-old vaccine is still administered due to lingering concerns regarding the use of aerosolised VEEV as a bioterrorism agent.

Here, we review recent advances in our understanding of how encephalitic alphaviruses spread among populations in the Americas and cause disease, as well as methods of diagnosis and treatment. We will also summarise the efforts to develop vaccines against these viruses using a range of platforms and current methods used to model infection of these viruses. This knowledge is invaluable for addressing bioterrorism threats associated with EEEV, VEEV, and WEEV and can be applied to control other climate-driven infectious diseases.

## 2. A History of Encephalitic Alphavirus Transmission: Phylogenetics and Epidemiology

### 2.1. Phylogenetics

The species under discussion in this review are all classified geographically as New World alphaviruses. EEEV, VEEV, and WEEV, collectively referred to as encephalitic alphaviruses, are characterised by encephalitic pathologies rather than the arthritogenic presentation common to many Old World alphaviruses—even those such as Chikungunya virus (CHIKV) now widely circulating in over 100 countries, including those in the Americas [18].

VEEV is notable among encephalitic alphaviruses for its high antigenic diversity, which confers subtypes with different properties of virulence, host compatibility and ecological distribution. Six VEEV subtypes, I-VI, are currently known [19]. Among them, subtypes II-VI are solely enzootic strains avirulent in equids, though their ability to infect humans remains intact, and there is no known difference in human disease course or sequelae between enzootic and epizootic strains [20].

High antigenic diversity is also observed within VEEV subtypes, with subtype I comprising 5 additional subtypes of diverse geographic origin, several of which are of considerable clinical significance. Strains ID and IE, originating in Colombia and Panama, are also enzootic in behaviour, but epizootic strains in the IA and IC subfamilies display significant equid transmissibility and high virulence in both equines and humans [21]. Strain IAB was responsible for major VEEV outbreaks until 1973, but in recent decades, strain IC has emerged as the primary driver of human VEEV epidemics [20,22]. Despite this, some research attention remains on strain IAB due to historical investigation of its use as a biochemical threat agent, owing to a particularly high readiness to aerosolise, and due to the large proportion of laboratory-acquired VEEV infections resulting from strain IAB aerosolisation [22].

EEEV displays significant genetic divergence across strains, which are separated by stark geographic and ecological differences. Four diverse EEEV lineages arose between 1960 and 2010. EEEV of North America exhibits such increased virulence in humans that the South American EEEV strains, which had not been associated with human outbreaks before the 2010 Darien outbreak, were ultimately reclassified to the species Madariaga virus (MADV) [19,23].

In contrast to EEEV, WEEV retains a higher degree of genetic similarity between North and South America, sharing >90% nucleotide identity in some coding regions [24], although this study was conducted before the recent resurgence of WEEV in South America. WEEV, a natural chimaera of EEEV and the Old World Sindbis virus (SINV) [25,26], shows limited evidence of drift towards decreased virulence in the modern B3 lineage now dominant in North America [27,28,29], though, likely, differences in apparent virulence from older A, B1 and B2 sublineages may have alternative ecologically and diagnostically driven explanations [29,30]. Analysis of the 2023–24 WEEV outbreaks proposes the emergence of a distinct C lineage in South America [31], which has evolved independently from North American strains for decades and has higher apparent virulence in humans, though further studies of these isolates and outbreaks are required to understand phylogenetically derived geographical differences in WEEV characteristics.

### 2.2. Transmission

**Figure 2:**
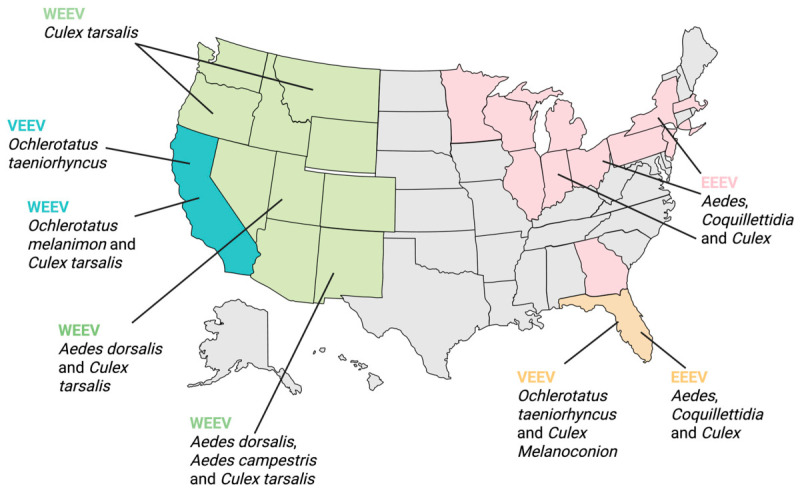
A map illustrating the distribution of mosquito species that act as vectors for encephalitic alphaviruses [20,24].

**Figure 3:**
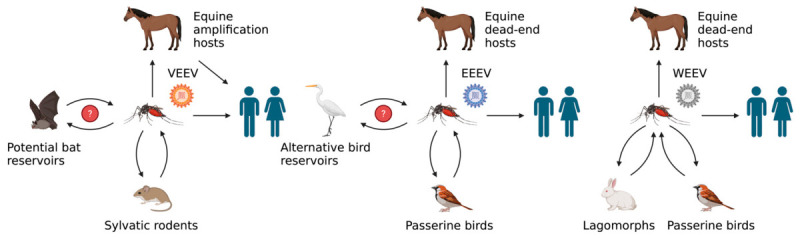
Transmission cycles of VEEV, EEEV and WEEV. Dead-end hosts are defined as those who do not sustain sufficient viraemia to infect feeding mosquitoes [34].

Encephalitic alphaviruses belong to a family of arboviruses that are primarily transmitted by mosquito vectors (Figure 2), except the Bijou bridge strain of the VEEV complex, which may be transmitted by the insect *Oeciacus vicarious* [19]. However, the role of an increasing range of species within enzootic cycles, acting as amplification hosts capable of producing sufficient viraemia for onward mosquito transmission, remains under investigation [32,33]. A summary of these transmission cycles is depicted in Figure 3. Direct transmission from horses is likely to occur in some epizootic cases, as the viruses can be shed in bodily fluids and transmitted through direct contact or aerosolisation. However, except for VEEV, equines are considered dead-end hosts for onward mosquito-vectored transmission, due to insufficient viraemia. Humans are likewise regarded as dead-end hosts for the same reason [34].

VEEV can be transmitted by several mosquito species, with enzootic cycles supported by *Culex* species and *Aedes taeniorhynchus* acting as the primary bridge vector for epizootic outbreaks [24,35]. Enzootic strains circulate amongst sylvatic rodents, with major reservoirs belonging to the *Peromyscus*, *Oryzomys*, *Proechimys*, *Zigodontomys* and *Heteromys* genera [19]. Transmission model research has characterised viraemia and successful disease-free seroconversion within spiny rats (*Proechimys* spp.) and cotton rats (*Sigmodon hispidus*), though the observed variance between enzootic and epizootic strains indicates a role for allopatric speciation and resistance selection in rodent transmission cycles [32,36,37]. There is also serological evidence that several bat species, especially from the genus *Artibeus*, may experience routine VEEV infections, though their involvement as a maintenance host is not fully understood [19,34,38]. Lastly, equids play a well-documented role in amplifying the epizootic strains of VEEV [24], serving as a reservoir species with rapid infectivity rather than a dead-end host as in EEEV.

EEEV transmission is also supported by multiple mosquito species, with enzootic cycles between passerine birds and the ornithophilic mosquito *Culiseta melanura* [39]. Bird models of transmission are especially relevant for EEEV, for which it has been proven that house sparrows (*Passer domesticus*) can support high viraemia of EEEV and MADV [39], albeit with some increases in mortality for EEEV, showing the role of species in symptomatic presentation. Snowy egrets have also been explored for EEEV, with proven ability to transmit to *Aedes albopictus* following subcutaneous infection and development of high viraemia, informing the range of mosquito species known to be viable for transmission [40]. Several mosquitoes serving as bridge vectors to epizootic equine and human outbreaks include *Aedes*, *Coquillettidia* and *Culex* species [39], though, notably, equines do not serve as amplification hosts, nor contribute to transmission dynamics [24,41].

Like EEEV, endozootic WEEV transmission can be maintained in birds, while human spillover is facilitated by multiple mosquito species in the *Culex* and *Aedes* genera [24,30,42]. However, lagomorphs are also major WEEV reservoirs, and, considering the 2023–24 outbreaks [31], the role of vector and reservoir species abundance within modern ecosystem transmission dynamics of WEEV remains worthy of study.

Animal models have also provided valuable exploration for encephalitic alphavirus transmission routes beyond the mosquito vector. Aerosol transmission, especially of VEEV, is of considerable research interest, and many mouse and non-human primate (NHP) studies have investigated this, though measurements of respiratory capacity and plaque assays of impaction samples are necessary to ascertain the administered dose [43,44]. EEEV aerosol exposure has demonstrated dose-dependent mortality in BALB/c mice and produces clinically relevant encephalitic presentations [45]. However, the lethality, measurability and accuracy of symptom recapitulation vary across virus, animal species, and model detail. As a result, universal conclusions cannot be drawn, particularly given the limited number of well-studied human aerosol transmission cases available for comparison. Also of interest is vertical transmission, as increased rates of abortions and central nervous system (CNS) birth abnormalities have been observed in infants born to mothers infected with VEEV during pregnancy [24]. Indeed, a study in rhesus monkeys showed vertical transmission of VEEV, with viral titres from the brain and uterus indicating infection and 67% of births displaying hydrocephalus, cataracts, and congenital microcephaly [46]. Congenital neurodevelopmental effects have also been observed in rhesus models of WEEV [47]. Further investigation of vertical transmission in encephalitic alphaviruses is therefore of significant clinical relevance.

## 3. Epidemiological History

### 3.1. VEEV

**Figure 4:**
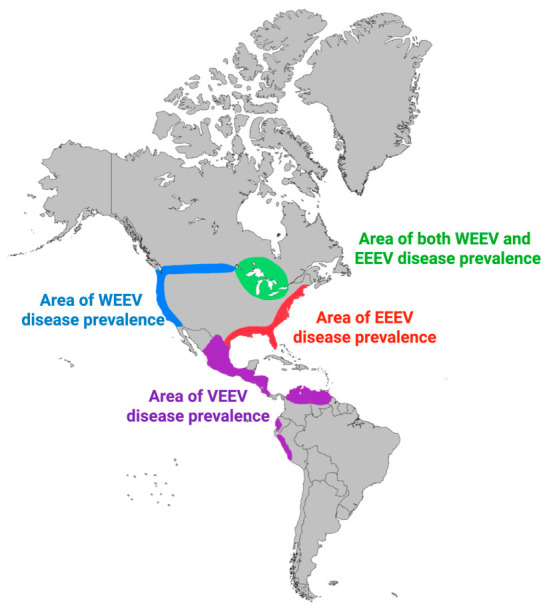
A map illustrating regions where WEEV, EEEV and VEEV disease are primarily found. These regions are primarily within North, Central, and South America [24,48].

VEEV has a range from northern Brazil, throughout Central America and into the southern states of the USA, primarily Texas (Figure 4) [24]. The virus was first isolated from an infected horse in 1938, though it had caused well-documented equine outbreaks in Colombia as early as 1935 and subsequently Venezuela and Trinidad, with epizootic outbreaks also reported in Peru from 1942 [19,20]. Significant VEEV outbreaks occurred throughout the 1960s, with one of the largest human outbreaks reported in Colombia and Venezuela in 1962, where the Venezuelan epidemic involved 23,283 documented human cases, of which 960 presented neurologically, and 156 were fatal [20,48]. The furthest-reaching series of VEEV epidemics occurred in 1969, with over 30,000 cases and 310 deaths documented in Ecuador at the start of the year, followed by outbreaks in Guatemala and El Salvador that expanded to 52,000 cases spanning most of Central America and reaching as far as southern Texas [19]. VEEV then entered a period of epidemiological silence until sporadic outbreaks occurred in 1992–1993, with another major outbreak in Venezuela and Colombia during 1995, in which as many as 100,000 human cases incurred 3000 cases of chronic neurological complications and 300 deaths [19,20].

VEEV epizootics do continue to occur, with several hundred cases fitting the VEEV symptom profile seen in a localised Colombia outbreak in 2008, though the virus itself was not isolated [19]. This is emblematic of issues with VEEV epidemiological surveillance and diagnosis, where symptoms overlap with several tropical diseases, though most notably dengue, obscures the true scale and frequency of outbreaks, and molecular testing techniques for case confirmation are not always accessible. Peruvian surveillance efforts found that VEEV caused a similar number of dengue-like febrile cases to dengue itself [49], and it is estimated that up to 7% of all Latin American dengue cases [50], or as many as 10% of suspected urban dengue cases, may be attributable to VEEV [19,20]. Historical periods of epidemiological silence may have had similar levels of unreported VEEV circulation. VEEV prevalence, however, does still notably change in response to ecological factors and public health interventions, or lack thereof. The 1969 outbreaks were ultimately contained by both chemical and non-chemical vector control and extensive equine administration of the TC-83 vaccine, with over 8 million doses issued [19]. Such methods have led to lower incidence in Colombia in recent decades, but incomplete vaccination of farm working horses and flooding events remain risk factors for viral amplification and vector proliferation, respectively. Novel vector control methods, such as gene drives and Wolbachia intervention, may prove relevant to ongoing VEEV transmission, though these methods remain to be tested on the complete range of VEEV vector species [51].

### 3.2. EEEV

EEEV circulates enzootically in eastern North America, especially in swampland regions, and periodically circulates in the Caribbean (Figure 4). Unlike VEEV, outbreaks are very limited in scale, and have been throughout the disease’s history, though like VEEV, it is thought that cases go significantly underestimated, with some investigations suggesting as few as 4–5% of infections are reported, especially in cases of milder presentation [52]. However, low case numbers are likely due to limited human interactions with the swamps where the disease is endemic, and the lack of equine amplification as a contribution to transmission dynamics [24,41].

Statistics are most comprehensive starting in 2003 with the CDC ArboNET initiative, which has recorded an average of 11 human cases per year within the USA, totalling 215 from 2003 to 2024, with the majority contracted in summer months of high vector abundance. Of these, 212 were neuroinvasive, and 84 were fatal, reflecting both the extreme virulence of EEEV among encephalitic alphaviruses and the reporting bias towards severe cases [52]. Though many cases are sporadic, some show outbreak behaviours, with 38 human cases and 26 horse cases recorded in the US Northeast in 2019, of which 19 were fatal, and many survivors exhibited neurological complications [52]. EEEV surveillance efforts are increasing elsewhere, with 100 prospective human cases identified within Panama in 2010 [53], and recent research into real-time reverse transcription-polymerase chain reaction (rRT-PCR) techniques for surveillance programs of EEEV, MADV and VEEV in the region [54].

### 3.3. WEEV

WEEV has a large endemic range, spanning Canada, the western United States, Central America, and now parts of South America [43]. WEEV was originally isolated from an infected horse in California in 1930 [41]. These early documented outbreaks of WEEV in the 1930s–40s were the most significant, involving tens of thousands of equine cases and over 3000 human instances, but since then, reported cases in North America have become less frequent, with fewer than 700 confirmed cases between 1960 and 1994 [31,55] followed by a period of epidemiological silence, as the virus has not been reported within mosquito populations since 2008 [55]. Likewise, modern transmission of WEEV in Central and South America had been reduced to some isolated incidence in Argentina in 1996 and Uruguay in 2009, before its recent resurgence [31].

In 2023, WEEV re-emerged in Argentina and Uruguay, where it was responsible for a major outbreak of 2548 equine cases mirrored by the geographical distribution of 217 human cases, 12 of which resulted in death, indicating considerable virulence [31]. Phylogenetic analysis of the viral isolates proposed a new C lineage, with closely shared nucleotide identity to a 1958 strain from Argentina, suggesting that the evolution of South American strains occurred independently for several decades, and indicating that WEEV circulation was ongoing in periods of apparent epidemiological silence, with many cases likely gone unreported [31].

## 4. Clinical Presentation and Pathogenesis

### 4.1. Common Symptoms

Most cases of encephalitic alphavirus infection remain either asymptomatic or as a self-limiting illness. However, a small number of cases progress into neuroinvasive diseases, which not only carry a substantial fatality risk but also the potential for long-lasting neurological sequelae. Symptomatic EEEV, WEEV and VEEV cases all generally present with similar clinical features and have an incubation period ranging from 2 to 10 days [56]. These symptoms are mild and non-specific and include headache, fever, malaise, myalgia, nausea, and vomiting. This febrile illness can become neuroinvasive and encephalitic, in the case of EEEV, following a 5–15-day incubation period. Following neuroinvasion, fever rapidly progresses, which may be accompanied by altered mental state, extreme lethargy, photophobia, paralysis, respiratory impairment, seizures or coma. EEEV carries a 30% mortality rate, increasing to 50–75% for over 60s, as well as a substantial risk of permanent neurological deficit. WEEV infection is generally milder in comparison to EEEV infection, with a lower risk of encephalitis onset and a 3–15% mortality rate if neurological invasion occurs [57]. Approximately 30% of VEEV-infected patients experience some symptoms. These are generally mild, systemic symptoms such as fever, lethargy, headache, chills, myalgia, nausea, vomiting, dizziness and prostration, which often subside in a few days but may recur. Neurological symptoms are observed in 4–14% of VEEV cases, with a fatality rate of 10–25% if encephalitis develops [58].

### 4.2. Neurological Symptoms

Although rarer, neurological disease and neuroinvasive behaviours are hallmark features of encephalitic alphaviruses. The mechanisms of neuroinvasion likely differ between viruses, but many of the neurological signs during acute infections are similar, including confusion, drowsiness, photophobia, stupor, seizure, paralysis and coma [41]. However, the subsequent development of long-term neurological complications does vary, with different frequency of occurrence and sequelae. These complications can be attributed to a combination of factors, including the neuroinvasion itself, replication damage incurred by CNS infections, and host inflammatory responses [59].

**Figure 5:**
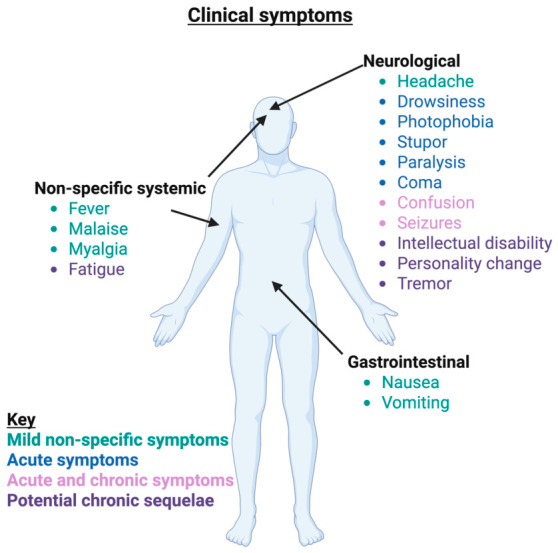
An illustration of the clinical symptoms of encephalitic alphavirus infection.

VEEV infection results in neurological complications among up to 14% (Figure 5) of surviving patients [41,43], although this risk is heightened for children, increasing to as much as 35% for under 5s [19,43]. These complications include convulsions, seizures, photophobia, hearing loss, anosmia, intellectual disabilities, altered personality, lingering confusion, fatigue, and depression [41].

EEEV infection has a 50–90% (Figure 5) chance of imposing neurological sequelae in survivors of neuroinvasive disease [41,43], whereas patients who showed no neurological symptoms during the acute phase of EEEV infection rarely suffer from neurological complications [43], a link less clearly seen for other encephalitic alphaviruses. Though EEEV sequelae are similar to those of VEEV, behavioural and memory alterations appear to be more common, and many survivors of the 2010 Panama outbreak developed chronic seizures associated with temporal lobe abnormalities [53]. As well as the expected encephalitic signs, histopathological EEEV studies revealed prominent cortical atrophy in people who died after extensive disease courses, many of whom developed cognitive and motor impairments after the acute phase, which is an observation of note for similar complications in survivors [60]. However, evaluation of survivors after rehabilitation suggests that EEEV-related complications may be reversible, as 40% showed sequelae improvement, increasing to 75% among those with the most severe complications [43].

WEEV also displays a high incidence of neurological complications, with 15–30% (Figure 5) of survivors developing significant permanent sequelae [24], rising to 50% among infants [43]. Historic WEEV case reports are a comprehensive source on complications ranging from sequelae typically observed in VEEV, to sequelae including tremor behaviours, anxious paranoia, and some occurrences of respiratory problems, including sleep apnoea and alveolar hypoventilation [43]. Some WEEV patients have neurological sequelae similar to patients diagnosed with Parkinsonism, and indeed the possibility of a relationship between WEEV and alpha-synuclein is currently under investigation [61,62].

### 4.3. Pathogenesis

While understanding in the area has progressed significantly, there is still no direct molecular or histopathological correlation recorded for specific encephalitic alphavirus presentations in humans [59]. The complexity of human disease and limitations in model systems have made it difficult to map mechanistic features directly to clinical symptoms. However, significant insights into viral behaviours, host immune interactions, and neuroinvasion mechanisms have emerged from animal models and experimental systems.

### 4.4. Viral and Host Determinants of CNS Pathogenesis

Murine VEEV models have been essential in exploring the relationship between inflammation and disease outcomes. Upregulation of mediators such as tumour necrosis factor-alpha (TNF-α), C-C motif chemokine ligand-2 (CCL-2), CCL-5, and leukocyte infiltrates appears to correlate with increased encephalitic pathology in the VEEV-Trinidad Donkey (TrD) model. Knockout (KO) studies also appear to reinforce this link, where increased survival times in KOs of inducible nitric oxide synthase (iNOS) and TNF-α have been observed, as well as reduced blood–brain barrier (BBB) permeability to VEEV in Toll-like receptor 4 (TLR4) KOs. Intercellular adhesion molecule-1 (ICAM-1) KO also shows reduced signs of neuroinflammatory damage [41]. Importantly, the role of immune-cell-mediated inflammatory damage is highlighted in VEEV-infected severe combined immunodeficient (SCID) mice, which exhibit spongiosis rather than encephalitis, displaying longer survival times and lack of hindlimb paralysis [63], implying that much of the pathology arises from immune-mediated inflammation rather than direct viral cytopathicity. However, immune responses are not always detrimental. A host immune response of sufficient magnitude to VEEV remains necessary for murine survival and disease resolution, as CD4+ T cells are shown to be essential for protection against lethality [64], and αβ-T cell receptor (TCR) KO mice showed persistent infection and elevated inflammatory infiltrates of the brain indicative of poor CNS viral clearance [65].

In comparison, less is known about molecular pathogenesis correlates in EEEV and WEEV animal studies. The tropism of wild-type NA-EEEV for heparan sulphate (HS) attachment receptors has been discovered, limiting proliferation of EEEV in murine lymphoid tissues and hence the febrile disease stage, while enhancing neurovirulence, likely contributing to the heightened neurovirulence properties of EEEV among encephalitic alphaviruses [66]. WEEV neuroinvasion is supported by caveolin-1 (Cav1)-mediated transendocytosis across intact BBBs, a method also employed by VEEV, which is abolished in Cav1 KOs where significantly decreased viral brain titres and encephalitic damage are observed [67]. Studies with WEEV also provide some support for the role of host inflammatory damage in encephalitic severity, as one study observed the peak of WEEV infection significantly preceded murine lethality, implying that host immune response also contributed to death as an outcome of infection [68].

### 4.5. Immune Evasion

Encephalitic alphaviruses use several strategies to evade the immune system which not only enables increased viral replication but also reduces vaccine efficacy. EEEV modulates the immune system to prevent an antiviral state from being established, whilst VEEV can resist the inhibitory effects [69]. nsP2 is a key protein in both these processes, inhibiting immune activation through several pathways. EEEV nsP2 degrades STAT1 and 2 to prevent type I interferon production through the JAK/STAT pathway. nsP2 further reduces interferon and cytokine production through disrupting IRF3 and NF-*κ*B nuclear translocation, enabling the virus to replicate and establishing a high-viral titre. Due to the peripheral immune evasion, eventual viral detection in the CNS leads to an excessive and imbalanced pro-inflammatory cytokine release, causing encephalitis [70]. Similarly, nsP2 is believed to play a critical role in immune modulation during VEEV infection. Unlike EEEV, VEEV infection triggers a high interferon *α*/β response and is resistant to the inhibitory effects of the antiviral state, notably replicating in interferon-primed cells [71]. Interferon *α*/β production is triggered following detection of VEEV RNA; however, this is rapidly downregulated. Viral replication produces nsP2 which induces host translational shutoff through its C-terminal domain. Translational shutoff is an effective immune evasion technique of VEEV through preventing host interferon-stimulated gene production, thus decreasing type I interferon *α*/β production and enabling viral replication [69].

As well as nsP2 activity, EEEV is believed to evade myeloid cells specifically through a binding site in the 3′ non-translated region. Myeloid cell-specific microRNA, miR-142-3p, binds to this 3′ region and suppresses viral replication. Suppressing viral replication prevents immune detection of the virus, thus reducing interferon *α*/β production. However, when mutated in this 3′ region, a significant increase in macrophage, NK cell, neutrophil and T-cell recruitment was seen, as well as a robust cytokine response. Thus, the miR-143-3p binding site is believed to prevent an adaptive immune response to EEEV infection [72]. Immune evasion motifs, such as those discussed here, should be avoided when designing a vaccine to induce a robust and useful immune response.

### 4.6. Host Factors Modulating Disease Outcomes

Direct insights into human pathogenesis itself, including risk factors and related correlates, remain limited. Infants and young children display heightened susceptibility to severe disease outcomes and neurological complications across encephalitic alphaviruses, likely due to age-dependent factors in adult neurons that are protective against viral CNS replication, such as apoptosis inhibitors conferring resistance to neuronal death [73,74]. It is also known that antibodies are an immune component highly significant to the clinical outcome severity of encephalitic alphavirus infections, though the neutralisation dynamics and efficacy are not fully understood in encephalitic alphaviruses, posing barriers to therapeutic monoclonal antibody (mAb) administration [75].

### 4.7. Animal Models of Pathogenesis

Due to these limitations in direct human study, animal models are indispensable for defining correlates of protection and elucidating disease pathogenesis. A range of models are chosen based on their ability to simulate specific aspects of human disease, including transmission, neuroinvasion, immune responses, and chronic sequelae.

Mouse models are the most widely used due to their genetic tractability and well-characterised immune systems, and particularly the well-characterised similarities in alphavirus pathogenesis, such as the importance of the olfactory system in neural invasion and age susceptibility to serious infection. Further to this, the restricted replication of EEEV in human leukocytes in vitro compared to VEEV mirrors observed weaker viral replication of EEEV in mouse macrophages and dendritic cells compared to VEEV [76]. Inbred C57BL/6 and outbred CD-1 mice have been particularly instrumental in studying encephalitic alphavirus pathogenesis in the contexts of both natural infection and biological warfare, and particularly following acute infections [44,76]. The key variables influencing infection outcomes include the inoculation route, viral dose, strain, genetic background, and age [45,67,77]. In particular, age has been shown to significantly impact susceptibility. Labrada et al. demonstrated that 14-day-old mice infected intraperitoneally with EEEV exhibited 100% lethality, while 6-month-old mice showed 80% survival. This increased survival was associated with stronger antibody responses and delayed CNS invasion, mirroring human outbreaks where infants under two are the most severely affected [78]. Similarly, studies in IFNAR−/− mice demonstrated uncontrolled viral replication in brain endothelial cells and pericytes, underscoring the role of type I interferons in neurovascular protection [67]. Additionally, BALB/c mice have been used to characterise VEEV TrD pathogenesis, which produces clinical signs like circling and head tilt and is strongly correlated with elevated TNF-α, CCL-2, CCL-5, and CD45^+^ infiltration [45], potentially identifying candidate biomarkers of encephalitic severity.

However, mice often show uniform susceptibility and rapid disease progression, limiting their use for studying long-term effects. For example, subcutaneous infection with EEEV results in near-100% mortality and CNS invasion as early as 1-day post-infection [79]. This is also true for VEEV, which causes 100% fatality within 7–10 days in mice despite being rarely fatal in humans [44,76]. Subsequently, refined models to study persistent human disease have been developed. Low-dose (10^3^ PFU) intranasal administration of the attenuated VEEV vaccine strain TC-83 in C57BL/6 mice produces persistent neurological impairment and thalamic damage [77]. Similarly, non-lethal VEEV infection has been achieved through recombinant mutants of the TrD strain in both CD-1 and C57BL/6 mice [44]. In WEEV models, passive immunotherapy using polyclonal antibodies against the E1 glycoprotein prevented lethal infection in CD-1 mice, allowing analysis of neurodegeneration even after viral clearance at 8 weeks [62].

Hamsters provide an alternative, more closely resembling human neuropathology such as vasculitis and microhaemorrhages, particularly following subcutaneous EEEV infection [44]. Guinea pigs, while being slightly more resistant, have been proven to be useful in modelling aerosol transmission and neuropathology and showed a higher incidence of encephalitis and coma than mice [43,44,80]. A drawback of the guinea pig and hamster models, as well as rabbit models, of VEEV, is the tendency for these animals to succumb before onset of CNS disease, making them of limited use [76].

Rats are less frequently used, although their larger brains and established use in neurobehavioral research can make them useful in studying VEEV-induced metabolic disruptions and foetal damage during pregnancy [44].

NHPs, particularly cynomolgus macaques, most accurately reproduce the clinical course of human encephalitic alphavirus infection, including a biphasic disease course [43,44]. For example, EEEV-infected macaques develop tremors, seizures, twitching, ataxia, head pressing, and nystagmus [81], while subcutaneous infection causes ataxia, tremors, and lethargy [82], highlighting how inoculation strategies affect disease course. These models are indispensable for vaccine and therapeutic development, especially in high-containment settings, despite ethical and logistical constraints. Despite their similarities in pathogenesis to humans, a disadvantage to NHP models of infection is the higher associated cost and logistical concerns, making them a less practical model, as well as lacking certain key markers of encephalitis, such as vasculitis, hemorrhage, and demyelination [76]. This makes NHP models less suitable for exploratory research and better suited for testing vaccines and therapeutics that have already showed promise in other pre-clinical models and are nearly ready for clinical trials.

While valuable for therapeutic and vaccine research, it is important to note that neurological models often rely on general health checks, which can miss key signs due to human interference. Implantable telemetry systems overcome this by enabling continuous, real-time monitoring of parameters like electroencephalogram (EEG), electrocardiogram (ECG), temperature, and activity without researcher presence [41]. In EEEV-challenged macaques, for example, telemetry detected circadian disruptions, reduced intake, and seizures that may have gone unnoticed [79]. Other promising improvements include expanding the use of telemetry to other models, incorporating neuroimaging and behavioural testing into small animal studies, and employing cDNA-derived virus strains to reduce attenuation artefacts to enhance model fidelity [20,41,81,83].

## 5. Diagnosis

### 5.1. Molecular Diagnostic Techniques

Symptoms incurred by encephalitic alphavirus infections are often non-specific; hence, definitive diagnosis relies on molecular or serological confirmation. RT-PCR is a common molecular method used for diagnosis [58]. Sánchez-Seco et al. developed a nested RT-PCR technique which amplifies fragments encoding nsP4. nsP4 is a highly conserved protein found in most alphaviruses, as it possesses RdRp activity. Through primers containing degenerated nucleotides at non-conserved positions, this nested RT-PCR can detect a broad range of alphaviruses [84]. Similarly, a multi-locus RT-PCR may be used. This technique uses multiple generic alphavirus sequence primers to detect a broad range of alphaviruses [85]. Despite these being useful techniques, several drawbacks are recognised. For example, sequencing is needed to identify the specific alphaviruses, which is associated with high labour intensity, requires specialised equipment, and is susceptible to sample contamination [54]. Alternative techniques such as real-time rRT-PCR have also been researched. One study by Carrera et al. designed separate probes for VEEV and MADV, each with a >95% alignment with their respective viral RNA sequences. When testing samples obtained from an outbreak in Panama during 2015–2017, these probes identified positive cases and detected the viruses in samples that had been previously thought to be negative. This illustrates the increased sensitivity of rRT-PCR alongside improved detection early on in infection, reduced labour intensity and quicker results compared to conventional RT-PCR [54].

### 5.2. Serological Diagnostic Techniques

Several serological methods can be used to detect encephalitic alphavirus infections, including haemagglutinin inhibition (HI) assays, complement fixation (CF), plaque reduction neutralisation tests (PRNT), enzyme-linked immunosorbent assays (ELISA), and immunofluorescence assays (IFA) [56,58,86]. More novel approaches also include nucleic acid sequence-based amplification (NASBA), RT-PCR, and TaqMan nucleic acid amplification assays [87,88]. Early sampling is critical as alphaviruses are recoverable from blood mainly within the first 2–4 days of illness, after which serology becomes more useful, with alphavirus-specific antibodies detectable in serum as early as one-week post-symptom onset [56,58].

Among these, IgM capture ELISA (MAC-ELISA) tends to be the most widely used for detecting recent infections. The peak detection for IgM antibodies normally occurs between one and three weeks, with levels declining within three months post-infection [56]. For confirmation, especially in regions where multiple alphaviruses co-circulate, PRNTs, which empirically measure functional nAbs, are considered the gold standard and offer higher specificity than ELISAs [56]. However, despite their accuracy, PRNTs present several practical challenges, including the requirement for highly trained personnel, CL-3 facilities, and several days to complete, limiting their routine availability [56]. Recent advances include the use of pseudotyped viruses and chimaeric SINV–VEEV constructs expressing VEEV structural proteins, which serve as safer alternatives to PRNT, HI, and CF assays without requiring CL-3 facilities [86,87].

In clinical settings, serological assays are especially valuable for identifying neuroinvasive disease, as the detection of virus-specific IgM antibodies in the cerebrospinal fluid (CSF) is considered strong evidence of CNS involvement. However, CSF-based testing is not typically used due to challenges with sample collection and a lack of standardised kits [56]. Instead, serological testing is more commonly performed on serum samples. Several reference laboratories in the United States offer IgM and IgG antibody testing for encephalitic alphaviruses, most frequently EEEV and WEEV [56], while suspected infections with other alphaviruses may require the transport of serum or CSF to public health laboratories for specialised testing.

Cross-reactivity between closely related alphaviruses, especially in regions where multiple of them co-circulate, limits the specificity of serological assays. This challenge is largely due to the E1 protein, a highly conserved structural protein that is a common target in diagnostic tests. For example, ELISAs alone often cannot reliably differentiate between alphavirus subtypes, necessitating confirmatory assays such as PRNTs or newer techniques like epitope-blocking ELISAs and microarrays capable of simultaneously detecting multiple viruses. A study of monoclonal antibodies (mAbs) from EEEV-infected patients illustrated this complexity, showing that some antibodies recognised EEEV by targeting domains I, II, and III of E1, while others broadly recognised several alphaviruses by targeting the domain II fusion loop of E1 [89]. Nevertheless, this study also demonstrated the successful use of recombinant E1 glycoproteins and virus-like particles (VLPs) as potentially safer and more scalable diagnostics [89]. Interestingly, it appears that E2, which is typically cross-reactive across arthritogenic alphaviruses such as CHIKV, O’nyong’nyong virus (ONNV), and Mayaro virus (MAYV), does not exhibit the same breadth of cross-reactivity with encephalitic alphaviruses [90]. As shown by Fox et al., a class of broadly neutralising mAbs targeting a conserved epitope on the E2 B domain, which was particularly effective across multiple arthritogenic alphaviruses, failed to bind cells infected with VEEV [65]. This was attributed to a greater genetic divergence of VEEV, which shares only 45.3% amino acid identity in E2 with CHIKV, lower than among the arthritogenic alphaviruses [90]. This highlights the potential utility of E2 diagnostics in distinguishing between more distantly related alphaviruses.

To address these limitations, paired acute and convalescent serum testing is recommended to demonstrate seroconversion and support a definitive diagnosis. Consideration of the semi-quantitative titre can help to differentiate between causative agents, as the virus with the highest titre is more likely to be associated with current disease [56]. Moreover, improved assays such as epitope-blocking ELISAs and alphavirus-specific microarrays are under development to increase diagnostic resolution. Structural protein microarrays, in particular, allow for parallel testing against multiple alphaviruses and may facilitate more accurate identification of the infecting virus, even in cases of co-infection or prior exposure. A recent study by Fischer et al. demonstrated that the use of parallel ELISAs and PRNTs significantly enhanced the ability to distinguish between co-circulating alphaviruses such as MAYV and CHIKV [91]. Since ELISAs alone may suffer from reduced specificity due to unknown degrees of cross-reactivity, incorporating PRNTs improves the diagnostic accuracy by confirming virus-specific nAbs. These findings highlight the importance of adopting multi-assay strategies in endemic areas, particularly as the geographic spread of arboviruses continues to expand due to increased vector range and international travel.

## 6. Treatments

### 6.1. Current Treatments

Currently, there are no publicly licensed therapeutics available for the treatment of infections by encephalitic alphaviruses. This limits clinicians to palliative and supportive care for infected patients. Management may include anticonvulsants to control seizures [92], painkillers for headaches, and rehydration for associated nausea and vomiting [93]. The lifetime cost of such care in the case of persistent symptoms and sequelae is often cited as upwards of USD 4 million [14,59]. However, this figure is based on findings from Villari et al. that are likely outdated and focused solely on the treatment of 13 patients suffering from EEEV infection. Accurate and up-to-date analysis of the costs of treatments in response to all three encephalitic alphaviruses is needed to truly appreciate the economic burden posed by the viruses and highlight the need for effective therapeutics [94].

### 6.2. Future Treatments

In the absence of effective treatments against encephalitic alphavirus infections, together with increasing concerns over a potential rise in cases and potential bioterrorism use, a wide range of novel therapeutic strategies are being researched. mAbs have been heavily researched, with many promising candidates. E1 and E2 are alphavirus surface glycoproteins hypothesised to be good anti-viral targets. They are involved in membrane fusion and receptor binding, respectively [89]. Williamson et al. analysed several mAbs, isolated from human EEEV survivors, which targeted domains I, II, and III of E1. Analysis showed that EEEV-346 targeted the domain II fusion loop of E1 and had strong pan-alphavirus cross-reactivity. However, a subcutaneous EEEV challenge showed poor protection with only 40% of mice surviving, compared to 10% of control mice. Another mAb, EEEV-138, was selected as it targeted the domain II fusion loop of E1, had similarly strong pan-alphavirus cross-reactivity, and showed high antibody-dependent complement deposition (ADCD). However, like EEEV-346, a subcutaneous EEEV challenge showed poor protection with only 30% of mice surviving, compared to 10% of control mice. Conversely, another mAb, EEEV-109, targeted the domain III of E1 and showed reduced pan-alphavirus cross-reactivity but provided 100% protection during the challenge [89]. In addition, another mAb, EEEV-179, recognised a quaternary epitope on E1 and resulted in 80% protection during the challenge. Similar studies have been conducted using anti-E2 mAbs against VEEV. These have shown 70% protection in mice following aerosol VEEV challenge, both when used prophylactically and post-exposure [95]. These studies have highlighted the potential for mAbs to be used as treatments against encephalitic alphavirus infections. However, further research into efficacy in humans is needed.

Drug repurposing strategies have the potential to be a timely and cost-effective way to discover anti-alphavirus treatments. Verwimp et al. gave alphavirus-infected fibroblasts a combination of three approved, antiviral oral nucleoside analogues: sofosbuvir, molnupiravir, and favipiravir. Combining molnupiravir and sofosbuvir suppressed all alphaviruses in the fibroblasts in a dose-dependent manner. This highlights how combining existing antivirals may serve as an effective treatment against alphaviruses [96]. Due to viruses utilising host machinery, identifying antiviral targets which are specific to the virus can be challenging. One such target is nsP4, a key protein found in many alphaviruses, with RdRP activity. Through in silico nsP4 modelling and molecular docking, Pareek et al. were able to identify four potential inhibitors. The viability of these compounds as antivirals was initially investigated through surface plasmon resonance to determine the binding specificity and a cytotoxic assay to ensure low cytotoxicity. CHIKV-infected baby hamster kidney-21 (BHK-21) cells were then given piperine, 2-thiouridine, pyrazinamide, or chlorogenic acid. Piperine and 2-thiouridine significantly reduced the viral load in CHIKV-infected BHK-21 cells, with similar results seen in SINV-infected cells, suggesting that it may also work against a range of alphaviruses, including encephalitic forms. This not only illustrates the potential of in silico modelling for the discovery of new treatments but also illustrates the potential of piperine and 2-thiouridine as alphavirus antivirals [97]. Many other drug mechanisms have been researched, such as anti-CD137 mAbs [98], interferon-alpha injections, and antisense oligonucleotides [99]. These highlight the great progress in the field and provide hope for an effective treatment against encephalitic alphaviruses in the near future.

## 7. Current Vaccine Trials and Development

### 7.1. VEEV—Live-Attenuated Vaccines

The use of live-attenuated viruses to protect against disease is a historically long-standing practice due to the limited techniques required to produce attenuation, exemplified by the process through which TC-83 was developed [17,100]. This vaccine, alongside a formalin-inactivated version named C-84, is still in use under the IND classification, with C-84 being used as a booster vaccination and for non-responders following failure to provide protection in animal challenge models [101,102,103,104]. Despite their weak immunogenicity profiles, the fact that close to 1/5 of TC-83 recipients are non-responders, undesirable adverse effects [102], and the potential implication of TC-83 being the origin of a VEEV outbreak in the late 1960s/early 1970s [104], these vaccines are still in use today for at-risk workers, such as those in laboratories or in the military. Overall, safer and more effective vaccines than the current ones available to at-risk workers are needed, and the development of vaccines suitable for public licensing is crucial.

One such attempt to reduce the reactogenicity of a live-attenuated vaccine involved mutation of the furin cleavage site between structural proteins E2 and E3. A candidate live-attenuated vaccine, V3526, was developed via site-directed mutagenesis of V3000, a cDNA clone of the TrD strain of VEEV [105]. Despite promising results from testing in mouse and horse animal models [105,106], a phase I clinical trial of V3526 (NCT00109304) was withdrawn due to unacceptable adverse effects, consisting primarily of fever and flu-like symptoms [107]. Inactivation of this vaccine has been attempted to improve safety through both formalin and 1,5-iodonaphthyl azide routes (F-iV3526 and INA-iV3526, respectively). However, these inactivated vaccines were poorly characterised and were not developed further [108,109]. Recently, V3526 has been mutated within the RdRp region to produce weaker-replicating mutants, referred to as 3X and 4X, mirroring an approach attempted in TC-83. These mutants appeared to have similar immunogenicity or tissue tropism, but the paper neglected to assess mouse reactions through febrility, activity, and higher-resolution telemetry data, which would be invaluable in avoiding a repetition of the results of the V3526 phase I clinical trial. Additional caveats of the 3X and 4X mutants include reversion back to the parental strain within five passages in cell culture for both vaccines [110]. This highlights a need to further stabilise these vaccines if they are to be adapted for future use.

### 7.2. VEEV—Nucleic Acid Vaccines

The use of genetic materials to induce antigen expression in host cells and elicit an immune response is an attractive strategy, whether through DNA- or RNA-based vaccine administration. A primary advantage of these approaches is that they do not require repeated in vitro passaging, which is often required for live-attenuated vaccines and may lead to reversion to virulence. Further to this, these vaccines can be easily manipulated, and there are much fewer safety risks surrounding their use. The use of DNA over RNA provides some advantages, as DNA is more stable and therefore does not need to be kept in the same low temperatures as RNA, potentially making it more suitable for widespread use in economically disadvantaged areas and in rural areas.

One such DNA vaccine, pWRG/VEE, is formed from a pWRG7077 plasmid backbone expressing codon-optimised structural proteins of VEEV, omitting the capsid protein due to its cytotoxicity and reduced transcription [10]. After animal testing through intramuscular routes followed by electroporation [111,112], a phase I clinical trial (NCT01984983) demonstrated acceptability and reactogenicity in humans [113]. While promising, this has not been progressed to a phase II trial, likely due to logistical issues surrounding electroporation use in these disadvantaged areas, the primary one being access to suitable power sources and transport of the machinery. To combat this, a novel administration method, jet injection, was tested in NHP models using this DNA vaccine. Intramuscular jet injection of pWRG/VEE was found to show promising results in NHP models [114], and a phase I clinical trial has been completed testing the administration of a candidate DNA vaccine in humans via jet injection (NCT06002503). A key advantage of this method is that the guns themselves are manually operated via a spring that the administrator can pull back to prime the gun, and electroporation is not required.

The pWRG/VEE vaccine has been developed further, with monovalent counterparts for EEEV and WEEV being combined into a trivalent vaccine [112]. While fully protecting mice from viral challenges against all three viruses, the trivalent vaccine typically induced lower NTs compared to the monovalent counterparts. Further to this, it induced a weaker humoral response than TC-83 in mice and a weaker cellular response than the monovalent VEEV counterpart. In rabbits, the humoral response was overall comparable between the trivalent and monovalent vaccines, but boost vaccinations appeared less effective, resulting in decreasing responses over time. This could be in part due to immune interference between the three mixed monovalent vaccines.

Infectious DNA, or iDNA, has the potential to revolutionise the field of vaccinology by combining the advantages of DNA and live-attenuated vaccines. Initially, a plasmid encoding the full RNA genome of TC-83 (pTC-83) was tested, using electroporation to administer the vaccine. This resulted in in vivo synthesis of the attenuated TC-83 virus capable of replication [115]. This plasmid has since been adapted, rearranging the structural cassettes to place the capsid protein after the rest of the structural proteins, as structural gene rearrangement has previously been shown to alter immunogenicity and reactogenicity in other viruses [116,117]. They also secured a key mutation on TC-83 by making a synonymous mutation, resulting in the codon coding for the same amino acid, but being two mutations away from the original TrD amino acid in that position. This plasmid, pMG4020, was shown to make V4020 in vivo in mice. Both the DNA vaccine and live-attenuated virus produced in culture can be used to vaccinate, with as little as 500 ng of DNA being protective [118]. pMG4020 has been demonstrated as safer and more stable than TC-83 [119], while V4020 is immunogenic and safe within macaques, protecting against 10^7^ plaque-forming units (PFUs) by aerosol challenge when administered intramuscularly [120].

More recently, alternative administration methods have been employed to improve ease of administration and, primarily, to avoid the use of electroporation for DNA vaccines. The use of microneedles has been tested for administration of both pMG4020 and V4020 in rabbits, and full seroconversion was observed, as assessed by PRNTs [121]. Further characterisation of these promising responses in other animal models through microneedle administration is required to ensure safety and efficacy. Comparisons between microneedles and jet injection, should both be proven successful, can inform the development of a revolutionary approach to roll out vaccination programs in remote and rural areas at risk of outbreaks.

### 7.3. VEEV—MVA Vaccines

Use of an attenuated Modified Vaccinia Ankara (MVA) strain, MVA-BN, to express viral polyprotein-encoding cDNAs has allowed the formation of monovalent and trivalent MVA vaccines against encephalitic alphaviruses. Primarily examined by the subcutaneous route, both the triple mix and trivalent vaccines showed a lack of neutralising titres (NTs) against VEEV and reduced titres against EEEV and WEEV, as compared to the monovalent vaccines [122]. These vaccines have been progressed to testing in mouse challenge models with both homologous and heterologous strains. All monovalent, trivalent, and triple mix vaccines protected the mice against homologous and heterologous challenges. The triple mix vaccine appeared to have an overall lower immunogenicity profile than the trivalent vaccine, which showed responses comparable to the monovalent vaccines. During assessments of temperature and body weight, no adverse effects within the mice were detected. Some abnormal behaviours were reported by the authors, but this was said to resolve [122].

A recent phase I clinical trial of MVA-BN-WEV (a trivalent vaccine) demonstrated higher immunogenicity than TC-83, as assessed by seroconversion, but also resulted in various flu-like symptoms in as many as one third of participants. While the authors still reported an acceptable safety profile, the inability of TC-83, with approximately 40% incidence of flu-like symptoms, to get officially licensed for public use indicates that this vaccine is unlikely to progress without additional attenuation to mitigate these adverse effects. Furthermore, after 32 weeks, only the highest-dose group appeared to retain a reasonable immune response [123].

### 7.4. VEEV—VLP Vaccines

The use of VLPs has permitted many advances in how we develop vaccines. Firstly, their replication deficiency greatly improves reactogenicity in those administered with the vaccine and helps keep laboratory workers safe when producing the vaccines. There is no concern for reversion to virulence, and the immune system still encounters a 3D particle presenting the same structural proteins, making immune responses generated more physiologically relevant to the real virus challenge.

The VRC 313 trivalent VLP against all 3 encephalitic alphaviruses was produced through transfection of human embryonic kidney 293F (HEK293F) cells with C-E3-E2-6K-E1 eukaryotic expression vectors for the relevant virus [124]. Mutations of the nuclear localisation signal were seen to result in the improved production of VLPs, and the icosahedral spike arrangement resembling that of the wild-type virus has been confirmed with electron microscopy. This has been tested in BALB/c mice and NHP models through intramuscular prime-boost regimens, followed by viral challenges. Full survival against viral challenge was observed, as assessed by viremia for VEEV infection of NHPs and survival in all other conditions. Brain pathology also showed a marked decrease in haemorrhaging and inflammation in the vaccinated as opposed to the control group following challenge.

A phase I clinical trial testing VRC 313 in different doses alongside an aluminium hydroxide adjuvant (NCT03879603) was conducted in 2021 [94], reporting no fever from any patients. While the 30 mg + adjuvant dose was significantly more immunogenic than without the adjuvant, as assessed by PRNT_80_, there were no significant differences between 30 mg + adjuvant and either of the 60 mg conditions. However, after 36 weeks, most responses had dropped off, and even the strongest was only marginally above 1:10, while a PRNT_80_ of 1:20 is often considered adequate protection. This suggests a lack of desirable long-term protection, in contrast to a VLP vaccine against CHIKV that induced high NTs in humans at least 2 years post-vaccination [125]. This difference may reflect unknown consequences of mutations within the capsid protein NLS in the VRC-313 vaccine, whereas the CHIKV VLP vaccine included only the capsid, E1, and E2 proteins derived from the Senegal strain. Alternatively, VRC-313 may perform worse because of immune interference, a phenomenon observed in simultaneous administration of neurotropic alphavirus vaccines [126].

A separate VLP vaccine excluding the capsid and including a mutation in the furin cleavage site has also been tested in pre-clinical models [127]. Three monovalent vaccines were tested as well as a mixture of the three, “V/W/E”. A mouse challenge study showed complete protection by all vaccines. However, the V/W/E vaccine appeared less immunogenic compared to the monovalent vaccines, as suggested by the IgG titre. Most vaccines exhibited borderline responses at 28 days post-prime and developed stronger responses by the 2 weeks post-boost timepoint, with full protection being observed 1 month post-boost. Twelve months post-prime, the monovalent vaccines still displayed 100% protection from viral challenge. V/W/E, however, while still being highly protective, did not provide full protection by this point. Notably, the monovalent VEEV vaccine also afforded cross-protection to other VEEV strains, with nearly 100% heterologous protection 12 months post-prime.

In an NHP challenge study, VEEV induced 100% seroconversion in macaques, while EEEV and WEEV were not as immunogenic, and seroconversion was not complete. The incomplete protection afforded by the vaccines is further exacerbated by the 50% survival rate in mock vaccinations for WEEV and EEEV, demonstrating that survival is not necessarily due to the vaccine.

This vaccine has recently been followed up on by Burke et al. [128]. However, this work focused primarily on establishing differences in vaccine administration routes, and an inappropriate animal model appeared to have prevented any solid findings.

A phase I clinical trial testing a monovalent VEEV VLP vaccine is also registered (NCT03776994). However, its status is currently “unknown”. The details of this vaccine and pre-clinical data are also not readily available.

### 7.5. VEEV—Other Vaccines

While many vectored vaccines against encephalitic alphaviruses have been developed and tested pre-clinically, there have, to our knowledge, been no significant advances in these vaccines in recent years, and previous development of these vaccines is well-reviewed elsewhere [15].

### 7.6. WEEV—Inactivated Vaccines

Similarly to VEEV, no vaccines for WEEV are currently available for public use. However, the vaccine TSI-GSD 210 is available to at-risk workers under the IND classification. TSI-GSD 210 was produced through formalin-inactivation of the virus passaged through chicken embryo cell culture, followed by freeze-drying [129]. Primary vaccination response rates of TSI-GSD 210 have been reported to be as low as 58%, with immune interference from simultaneously administered alphavirus vaccines further dampening responses [126], highlighting the need for more immunogenic vaccines.

### 7.7. WEEV—DNA Vaccines

The DNA-WEEV vaccine is developed from the same principle as the pWRG/VEE vaccine, a pWRG7077 plasmid expressing codon-optimised WEEV structural proteins E3-E2-6K-E1, similarly excluding the capsid protein [112]. In mouse models, delivered intramuscularly via electroporation, the DNA-WEEV vaccine fully protected against aerosolised WEEV of the same strain. In contrast, the TSI-GSD 210 vaccine only protected 30% of the mice from virus challenge. The mean PRNT_80_ reached 100 by 42 days post-prime in mice, while in rabbits, the PRNT_80_ approached 10,000 and remained high for 350 days post-vaccination. The results from the rabbit immunogenicity study, in particular, are encouraging for the development of the vaccine.

### 7.8. WEEV—MVA Vaccines

The MVA-BN-W vaccine conferred complete protection in mice against intranasal WEEV challenge [130], as confirmed by follow-up studies [122]. While the latter study also demonstrated a rise in NTs, a PRNT_50_ was calculated, while PRNT_80_s are typically used to predict the level of protection.

### 7.9. WEEV—VLP Vaccines

A constituent of the VRC-313 vaccine, the WEEV VLP vaccine consists of the full structural protein cassette of WEEV, with an NLS mutation to improve expression of proteins, particularly the capsid protein [124]. The vaccine demonstrated complete protection in both mouse and NHP models, also exhibiting strong humoral responses, as assessed by PRNT_80_. In the mouse models, however, the WEEV VLP vaccine appeared less immunogenic than the other monovalent vaccines. Further to this, the duration of protection is not well-assessed, with humoral immunity not being tested beyond 49 days post-prime.

The WEEV-GP VRP, a monovalent VRP vaccine and constituent of the V/E/W VRP vaccine, demonstrated complete protection against viral challenge in mouse models from 2 months post-prime, lasting at least until 12 months post-prime. In an NHP model, however, only near complete protection was afforded, and PRNT_80_s were typically lower than other monovalent vaccines. Overall, as a monovalent vaccine, it appears to lack sufficient protection and humoral responses. Cellular responses were not assessed [127].

### 7.10. WEEV—Other Vaccines

While many other vaccines against WEEV have been developed and tested pre-clinically, there have, to our knowledge, been no significant advances in these vaccines in recent years, and the previous development of these vaccines is well-reviewed elsewhere [15].

### 7.11. WEEV—Clinical Trials

Aside from the phase I clinical trial for the trivalent VRC-313 VLP vaccine, the only clinical trials for WEEV vaccines, to our knowledge, are continued investigations into TSI-GSD 210 carried out by the US Army at Fort Detrick, Maryland. These consist of a phase II trial currently in “Active—Recruiting” status (NCT02466750) following a relatively successful phase I trial (NCT01159561). While this phase I trial demonstrated high response rates, considering a PRNT_80_ > 40 as a positive response, this was following three primary vaccinations in a small group size, and even fewer members of the original cohort appeared to be included in the 6-month follow-up [131]. Despite the low rate of systemic adverse events observed for this trial, it cannot be ignored that two other older phase II clinical trials described in the same paper demonstrated much higher rates of adverse effects and strikingly less effective response rates. While the authors suggested this could be due to differences in lots, all lots passed required testing, and this vaccine is not, in its current state, suitable for licensing. 

### 7.12. EEEV—Inactivated Vaccines

Efforts to develop a human vaccine for EEEV have been ongoing for over 80 years, with renewed momentum in recent decades due to concerns over increasing outbreak frequency and the virus’s potential as an aerosol-transmissible biothreat. Yet, there is still no licensed human vaccine currently available, although many attempts have been made with many current developments underway (Table 1). Initial work by the U.S. Department of Defence in the 1940s led to the development of a formalin-inactivated chicken embryo vaccine (PE-6 strain), which, despite demonstrating nAb responses in most recipients, required multiple booster doses to maintain immunity [126]. The product, later designated TSI-GSD 104, achieved an 84% nAb response rate after a two-dose primary series and intradermal booster, but protection was transient and required frequent re-administration [132]. Although this inactivated vaccine remains available under the IND protocol for military and laboratory use, its poor immunogenicity and batch-to-batch variability have hindered broader deployment [132]. Moreover, as with other inactivated viral vaccines, concerns about incomplete inactivation and epitope distortion remain unresolved [133].

**Table 1:** Encephalitic alphavirus vaccines.

Virus	Vaccine Platform	Vaccine	Description	Development Stage	Efficacy	Advantages	Disadvantages
VEEV	Live-attenuated	TC-83 [17,100]	Attenuated via multiple passages in chicken embryo cell culture	Used for research and at-risk workers under the IND protocol	Partial protection, incomplete seroconversion	Easy to produce by attenuation	Undesirable adverse effects, incomplete seroconversion, risk of reversion to virulence
		V3526 [105,106,107]	Site-directed mutagenesis of the V3000 strain (TrD), primarily in the E2-E3 furin cleavage site	Phase I clinical trial (NCT00109304) withdrawn due to adverse effects	Effective in animal models. However, human trials stopped due to febrile symptoms	Reduced reactogenicity	Adverse effects in humans, poor safety profile
		V3526 3X and 4X mutants [110]	RdRp mutated variants of V3526	Pre-clinical, mouse model	Retained immunogenicity in mice	Potentially safer replication, stable tissue tropism	Reversion to the parental strain after passages, incomplete safety data
		V4020 [118,119,120]	Live-attenuated vaccine produced from the pMG4020 iDNA vaccine	Phase I clinical trial ongoing (NCT07088822); tested in mice and NHPs	Protective against subcutaneous VEEV challenge in mice and NHPs	Genome rearrangement conferred reduced reactogenicity compared to TC-83	More stable than TC-83 due to stabilising mutations. Further characterisation needed. Cannot be stored for as long as iDNA, especially in warm climates
		V4020 administered via hollow microstructured transdermal system (hTMS) [121]	V4020 administered transdermally via microneedle	Pre-clinical, tested in rabbits	Caused seroconversion in rabbit, assessed by PRNT_80_	Improved ease of use as electroporation is not required	Poorly characterised, only used in immunisation study in a rabbit model; only one rabbit immunised transdermally with the microneedle
	Inactivated	C-84 [101,102,103]	Formalin-inactivated TC-83	Used under the IND protocol as a booster for TC-83 or for TC-83 non-responders	Failure to provide protection in animal challenge models, used as a booster for TC-83	Safer than TC-83, can induce seroconversion in some TC-83 non-responders	Still a low overall seroconversion rate
		F-iV3526 [109]	Formalin-inactivated V3526	Pre-clinical mouse models	80–100% protection against VEEV TrD challenge	Near-complete protection when administered subcutaneously	No telemetric analysis performed in the mouse models to prevent a repeat of V3526
		INA-iV3526 [108,109]	1,5-iodonaphthyl azide-inactivated V3526	Pre-clinical mouse models	Similar to F-iV3526	Near-complete protection when administered subcutaneously	No telemetric analysis performed in the mouse models to prevent a repeat of V3526. Lower NTs than F-iV3526
	DNA	pWRG/VEE [111,112,113]	Codon-optimised structural proteins (capsid excluded) on the pWRG7077 backbone	Phase I clinical trial completed (NCT01984983)	Immunogenic in animals and humans	More stable than RNA, easily manipulated, less risk of reversion to virulence	Requires electroporation, challenging in remote areas due to the limited availability of power sources and transport of machinery
		pWRG/VEE administered via jet injection [114]	See pWRG/VEE	Phase I clinical trial completed (NCT06002503)	Promising results in NHP models	Electroporation-free, manually powered jet injection suitable for low-resource areas	Early stage, needs further validation
	iDNA	pTC-83 [115]	Full RNA genome	Pre-clinical; tested in mice	Protective against subcutaneous VEEV challenge in mice	Combines live-attenuated immunogenicity with DNA stability; safer and more stable than TC-83	Needs further validation for safety and delivery methods
		pMG4020 [118]	Rearranged genome of pTC-83	Pre-clinical; tested in mice	Full seroconversion assessed by PRNT_80_	Genome rearrangement conferred reduced reactogenicity	Plasmids require electroporation, challenging in remote areas due to the limited availability of power sources and transport of machinery
		pMG4020 administered via hollow microstructured transdermal system (hTMS) [121]	pMG4020 administered transdermally via microneedle	Pre-clinical, rabbit models	Full seroconversion assessed by PRNT_80_	Improved ease of use as electroporation is not required.	Poorly characterised. Only tested in immunisation studies in a rabbit model; only tested in 3 rabbits
	MVA	MVA-BN-V [122,130]	Codon-optimised E3-E2-6K-E1 protein expressed on an MVA-BN vector	Pre-clinical; mouse models	Full protection in mice against intranasal VEEV TrD	Induced NTs and interferon-gamma responses	Only tested in mouse models. PRNT_50_ used to assess nAbs as opposed to PRNT_80_
	VLPs	VEEV-GP virus replication particle (VRP) [127,128]	E3-E2-6K-E1 expressed from replicon (excluded E3-E2 furin cleavage site)	Pre-clinical; mouse and NHP models	Near-complete protection in mice; protection from viremia in NHPs	Long-term protection; reduction in febrile symptoms in NHPs	Weak immunogenicity in VEEV serotypes other than VEEV-IAB
		VEEV VLP [124]	C-E3-E2-6K-E1 structural proteins with NLS mutation. Constituent of VRC-313	Pre-clinical; mouse models	Complete protection in mice from challenge with aerosolised VEEV. Strong PRNT_80_ 49 days post-prime	Non-replicating; high safety; mimics the native virus; NLS mutation increases capsid protein expression	Poorly characterised, immunogenicity and protection over long periods not assessed. No models besides mice were used. Needs further studies for natural (mosquito) challenge; manufacturing scale-up required
		Undefined VEEV VLP	No information available	Phase I clinical trial completed (NCT03776994)	Not yet reported	Safe, stable, simpler manufacturing	Early stage; no information efficacy data pending
EEEV	Live-attenuated	EEEV IRES-modified [134]	Structural genes with EMCV IRES replacing promoter	Pre-clinical; mouse models	100% survival post-challenge; no viremia; no replication in mosquitoes	High immunogenicity; no mosquito replication; genetically stable	Live vaccine risks (mutation/reversion); NHP/aerosol protection not yet tested
		Rationally designed EEEV LAV [133]	Mutations in the 5′ untranslated region (UTR), capsid, E2, and 3′UTR	Pre-clinical (mouse; NHP studies proposed)	Near-complete protection against aerosol challenge (triple mutant)	Defined attenuation; strong T cell and cytokine responses; low reversion risk	Some mutants retain virulence; variable NTs; NHP validation needed
	Inactivated	TSI-GSD 104 [126]	PE-6 strain whole virus	IND (DoD use only)	60–84% seroconversion after primary + booster vaccination; protection not long-lasting	Safe, established; used in high-risk personnel	Weak immunogenicity; requires multiple boosters; risk of incomplete inactivation
		CVEV1219 [135]	Formalin-inactivated attenuated EEEV CVEV1219	Pre-clinical; mouse models	2 doses provided protection; formalin is the most effective of inactivation methods	Safer than the wild-type; can be combined with adjuvants	Inactivation may reduce epitope fidelity; aerosol protection inconsistent
	DNA	DNA-EEEV [112]	EEEV glycoproteins expressed via plasmid	Pre-clinical; mouse and rabbit models	Full protection in mice; 60% mortality in IND comparator	Electroporation enhances response; scalable	Requires prime + 2 boosts; inconsistent nAb responses
	MVA	MVA-BN-E [130]	Codon-optimised E3-E2-6K-E1 EEEV protein strain FL93-939NA expressed on an MVA-BN vector.	Pre-clinical; mouse models	Full protection in mice against intranasal EEEV	Induced strong NTs and cellular responses. Established technique	Only tested in mouse models. PRNT_50_ used to assess nAbs as opposed to PRNT_80_. Did not assess T-cell interferon-gamma responses. Incomplete seroconversion observed by Henning et al., despite full protection [122]
	VLPs	EEEV VLP [124]	C-E3-E2-6K-E1 structural proteins with NLS mutations. Constituent of VRC-313	Pre-clinical; mouse models	Complete protection in mice from challenge with aerosolised EEEV. Strong PRNT_80_ 49 days post-prime	Non-replicating; high safety; mimics the native virus; NLS mutations increase capsid protein expression	Poorly characterised, immunogenicity and protection over long periods not assessed. No models besides mice were used. Needs further studies for natural (mosquito) challenge; manufacturing scale-up required
		EEEV-GP VRP [127,128]	E3-E2-6K-E1 expressed from replicon (excluded the E3-E2 furin cleavage site)	Pre-clinical; mouse and NHP models	Complete protection in mice and near complete protection in NHPs; Up to 12-month protection	Long-term protection	Mild symptoms post-challenge in NHPs; no sterilising immunity
	Chimaeric alphavirus	SINV/EEEV [136]	SINV backbone + EEEV structural proteins (capsid, E1-E2)	Pre-clinical; mouse models	Survival after high-dose EEEV challenge; protection correlated with IgG seroconversion	Strong attenuation from chimaera; low reversion risk; no neurological signs post-vaccination	Some low-dose recipients not protected; cross-lineage efficacy untested; no NHP or human data
		SINV/EEEV Chimaera [137]	EEEV structural genes + SINV backbone	Pre-clinical; mouse and NHP models	82–100% survival; high NTs; no brain lesion	Live replication enhances immunogenicity; tested in the NHP aerosol model	Risk of recombination/reversion; further safety validation needed
		EILV/EEEV [138]	EEEV structural genes + Eilat virus (EILV) backbone	Pre-clinical; mouse models	100% survival; rapid seroconversion by day 6	Cannot replicate in vertebrates; excellent safety profile	No NHP or aerosol data; durability of immunity unknown
	Chimaeric vesiculovirus	ISFV/EEEV [139]	EEEV glycoproteins + Isfahan virus backbone	Pre-clinical; mouse models	100% survival post-challenge; single dose immunogenic	Single-dose potential; avoids vesicular stomatitis virus (VSV) cross-reactivity	Needs further testing in other challenge routes; limited data
	Subunit	E1-ecto LANAC [140]	E1 ectodomain (from WEEV) in liposome complexes	Pre-clinical; mouse models	90% protection 9 weeks post-boost; no nAbs	Cross-protective potential; non-replicating	Long time to achieve immunity; no early protection; low NTs
WEEV	Inactivated	TSI-GSD 210 [126,129,131]	Formalin inactivation of WEEV virus attenuated via passaging through chicken embryo cell culture	Used for research and at-risk workers under the IND protocol. Some clinical trials have recently been completed (NCT02466750 and NCT01159561)	Low seroconversion with response rates as low as 58%	Safer than live attenuated vaccines	Immune interference from simultaneous administration of other alphavirus vaccines can further dampen responses
	DNA	DNA-WEEV [112]	Codon-optimised E3-E2-6K-E1 WEEV proteins expressed via the pWRG7077 plasmid	Pre-clinical; mouse and rabbit models	Full protection in mice; only 30% survival for TSI-GSD 210 vaccinated mice.	High NTs up to 350 days post-prime	Requires electroporation
	MVA	MVA-BN-W [122,130]	Codon-optimised E3-E2-6K-E1 protein expressed on an MVA-BN vector	Pre-clinical; mouse models	Full protection in mice against intranasal WEEV	Induced NTs	Only tested in mouse models. PRNT_50_ used to assess nAbs as opposed to PRNT_80_. Did not assess T-cell interferon- gamma responses
	VLPs	WEEV-GP VRP [127,128]	E3-E2-6K-E1 expressed from replicon (excluded the E3-E2 furin cleavage site)	Pre-clinical; mouse and NHP models	Complete protection in mice from 2 months post-prime and near complete protection in NHPs; Up to 12-month protection	Long-term protection; Safer than live attenuated vaccines	PRNT_80_ 2 months post-prime significantly lower than that for EEEV-GP VRP
		WEEV VLP [124]	C-E3-E2-6K-E1 structural proteins with NLS mutations. Constituent of VRC-313	Pre-clinical; mouse and NHP models	Complete protection in mice from challenge with aerosolised WEEV. Strong PRNT_80_ 47 days post-prime in NHPs	Non-replicating; high safety; mimics the native virus; NLS mutations increase capsid protein expression	Poorly characterised, immunogenicity and protection over long periods not assessed. Weaker nAb responses than in EEEV and VEEV monovalent VLPs. Needs further studies for natural (mosquito) challenge; manufacturing scale-up required
V/W/E (trivalent)	DNA	3-EEV [112]	Mixture of DNA-WEEV, DNA-EEEV, and pWRG/VEE (DNA-VEEV)	Pre-clinical; mouse and rabbit models	Full protection against all 3 viruses in mouse challenge models	A multivalent vaccine, more stable than RNA vaccines, safer than live attenuated vaccines	Significantly lower NTs in rabbits compared to the monovalent VEEV vaccine. Overall weaker humoral and cellular responses in mice compared to monovalent vaccines. No significant improvement on TC-83
	MVA	MVA-BN-WEV [122,123,130,132]	Codon optimised E3-E2-6K-E1 polyproteins expressed together	Phase I clinical trial completed (NCT04131595); Phase II clinical trial ongoing (NCT06899802)	Higher seroconversion in humans than TC-83, but not complete. Induction of both humoral and cellular immunity. Protection appears lasting upwards of 6 months in high dose groups	No reported serious adverse effects in mice; potential for multivalent protection. No immune interference observed. An established technique	Only partial protection from VEEV and EEEV challenges in some mouse models. Lower NTs against VEEV compared to the other two viruses. PRNT_50_ used as opposed to PRNT_80_; Dose-dependent adverse effects in humans are likely still too high for it to be licensed without further attenuation; 1 SAE (pleural) possibly vaccine-related; Responses also began to decline approaching 32 weeks post-prime; EEEV cellular immunity not tested
		MVA-BN triple mix [122,130]	Three MVA-BN monovalent vaccines administered at once (MVA-BN-V, MBA-BN-E, MVA-BN-W)	Pre-clinical; mouse models	Partial protection against virus challenges. Incomplete seroconversion	No severe adverse effects reported in mice. Allows flexible dosing	The triple mix was proved to be less protective against EEEV challenge than the monovalent vaccine. Potential immune interference. The triple mix also had lower immunogenicity than the trivalent vaccine. Henning et al. only compared the triple mix to the trivalent and monovalent VEEV vaccines, not to the EEEV or WEEV monovalent vaccines. PRNT_50_ used as opposed to PRNT_80_ [122]
	VLPs	VRC 313 (trivalent VLP) [94,124]	Structural proteins C-E3-E2-6K-E1 with NLS knockout mutations	Phase I clinical trial completed (NCT03879603)	Full protection in NHPs and mice against viral challenges; Comparable immunogenicity to monovalent versions in mice and NHPs	No reversion risk, safer production, less reactogenicity, physiologically relevant antigen presentation	Short duration of immunity in humans, PRNT_80_ titres drop after 36 weeks, only marginally protective levels remained. Weaker nAb responses in mice to WEEV than to EEEV and VEEV
		V/E/W VRP [127,128]	A mixture of VEEV-GP VRP, EEEV-GP VRP, and WEEV-GP VRP	Pre-clinical; mouse and NHP models	Incomplete protection in mice challenge model for all 3 viruses. Incomplete protection from death/viremia in NHPs 12 months post-prime	Burke et al. showed promising protection and PRNT_80_ titres. However, this was for a much shorter period	Incomplete protection against three viruses. Responses were typically lower than those from monovalent vaccines

### 7.13. EEEV—Live-Attenuated Vaccines

A resurgence in EEEV vaccine research over the last two decades has led to the development of a wide array of platforms aimed at improving immunogenicity, safety, and scalability. Live-attenuated vaccines, historically effective in alphavirus immunisation (e.g., TC-83 for VEEV), are inherently more immunogenic but pose risks of reversion to virulence, especially when attenuation is conferred by only one or two nucleotide substitutions. This concern is particularly significant for EEEV, given its high mortality and neurotropism [135]. Consequently, modern live-attenuated vaccine strategies for EEEV, such as those incorporating an internal ribosomal entry site (IRES) element to restrict replication in mosquito vectors, have shown promise in mice with robust protection and no detectable viremia or brain infection [141].

### 7.14. EEEV—VLP Vaccines

VLP vaccines are another attractive option. These non-replicating constructs mimic native virions in structure but lack genomic RNA, thus avoiding reversion risks. An EEEV VLP vaccine demonstrated strong immunogenicity and complete aerosol challenge protection in NHPs, with no histopathological lesions, though further studies are needed to confirm efficacy following mosquito-borne infections [141].

### 7.15. EEEV—Chimaeric Vector Vaccines

Chimaeric vector vaccines represent a growing class of candidates that combine structural genes from EEEV with replication backbones from other viruses. For example, alphavirus-based chimaeras, such as SINV/EEEV or EILV/EEEV constructs, have conferred high levels of protection with favourable safety profiles, the latter being unable to replicate in vertebrate hosts [44]. These chimaeras may be particularly valuable in multivalent vaccines targeting multiple encephalitic alphaviruses. A chimaeric EEEV vaccine using the EILV backbone provided 100% survival in mouse challenge studies and showed rapid seroconversion, although long-term durability and efficacy in NHPs remain to be demonstrated [138].

### 7.16. EEEV—Subunit Vaccines

Subunit vaccines leveraging conserved structural proteins, particularly the E1 glycoprotein, have also been explored. Notably, although E1 is less immunogenic than E2, it is highly conserved and a major target of cross-reactive human mAbs, including pan-alphavirus mAbs that recognise EEEV, VEEV, WEEV, CHIKV, and MAYV. Several of these mAbs bind to the domain II fusion loop or quaternary epitopes of E1, some in a pH-independent manner, suggesting potential utility in both diagnostic and vaccine contexts [89]. However, subunit-based products may require adjuvants or delivery systems to enhance immunogenicity, and the time to protection may limit their utility in outbreak settings [44].

### 7.17. EEEV—Nucleic Acid Vaccines

Nucleic acid vaccines, including DNA and VRP approaches, have shown considerable pre-clinical success. EEEV VRPs induced long-lasting nAbs and provided complete protection in mice and macaques up to 12 months post-vaccination, though mild clinical signs were observed in some primates [44]. DNA plasmid vaccines delivered by electroporation also demonstrated survival benefit against aerosol challenge, though nAb responses were modest and required prime-boost regimens [141].

### 7.18. EEEV—Clinical and Regulatory Considerations

Despite this technological progress, no human EEEV vaccine has been advanced to licensure. Challenges include the rarity and geographic restriction of EEEV outbreaks, the absence of a predictable market, and high development costs, which are estimated at $500 million per product [133]. Nevertheless, the virus’s capacity for aerosol transmission, its potential use in bioterrorism, and the devastating neurological sequelae in survivors provide continued impetus for vaccine development, particularly within the context of trivalent vaccines targeting EEEV, VEEV, and WEEV.

### 7.19. Immune Correlates of Protection (CoP)

One of the major challenges in developing vaccines against encephalitic alphaviruses is the lack of well-defined immune correlates of protection (CoP). Although nAbs are widely considered the most likely correlate, no universally accepted protective threshold has yet been established for EEEV, VEEV or WEEV.

Many vaccine studies use PRNT assays to measure protective immunity, and higher nAb titres are generally associated with protection in animal challenge models. However, protection has occasionally been observed despite relatively modest antibody responses, suggesting that neutralising antibodies alone may not fully explain protective immunity. Cellular immune responses are also likely to contribute to protection. Studies of VEEV infection have demonstrated the important role of CD4+ T cells in viral clearance and survival, and several vaccine platforms induce both humoral and cellular immunity. These observations suggest that protection may depend on a combination of antibody and T-cell responses, rather than a single immunological marker.

While nAbs have been evaluated in vaccine recipients, there is remarkably limited direct evidence defining natural infection-induced protective immunity in human encephalitic alphavirus infection. This is most likely due to the sporadic nature of human disease, the limited availability of longitudinal cohorts from endemic regions, and the ethical constraints that preclude controlled human rechallenge studies [15]. This evidence gap contrasts with arthritogenic alphaviruses, for which natural infection has been more clearly associated with durable humoral immunity [142]. For example, natural CHIKV infection induces long-lived anti-E2 IgG responses, persistent memory B cell responses, and cross-neutralising antibodies against related arthritogenic alphaviruses within the Semliki Forest antigenic complex. However, this cross-reactivity appears largely complex-restricted and does not extend reliably to encephalitic alphaviruses, which are antigenically distinct [142].

Nevertheless, several sources provide indirect support for protective post-exposure immunity following encephalitic alphavirus exposure. The CDC reports that once infected with EEEV, individuals are thought to have lifelong immunity against EEEV reinfection, although this does not imply protection against other encephalitic alphaviruses [52]. Broader alphavirus immunology studies indicate that natural infection can induce antibodies against conserved E1 and E2 glycoprotein epitopes, including cross-reactive antibodies with potential protective relevance, supporting the concept of infection-induced humoral protection [142]. Data from the TC-83 live-attenuated VEEV vaccine, which partially mimics natural infection through limited viral replication, also suggest that durable protective immunity is achievable in some recipients. However, incomplete seroconversion, reactogenicity, and the use of C-84 boosting in non-responders indicate that replicating-virus exposure does not uniformly generate reliable or indefinite protective immunity [102,103,104].

Therefore, while available evidence supports the biological plausibility and likely success of prophylactic immunisation against encephalitic alphaviruses, current data are insufficient to define natural infection-derived correlates of protection, the duration of protective immunity, or optimal booster frequency. Booster schedules will therefore need to be determined empirically for each vaccine platform, ideally using longitudinal nAb titres, memory B cell responses, and challenge-model data linked to protection.

Identifying robust CoP is particularly important because the sporadic nature of encephalitic alphavirus outbreaks makes conventional efficacy trials difficult to perform. Consequently, future vaccine licensure may rely on regulatory pathways such as the FDA Animal Rule, where efficacy is inferred from well-characterised animal models and immunological markers that can be bridged to humans. A better understanding of protective immune responses is therefore essential not only for predicting vaccine efficacy and booster requirements, but also for accelerating vaccine development and regulatory approval.

### 7.20. Emerging Themes in Vaccine Development

There has been substantial progress in developing vaccines against encephalitic alphaviruses (EEEV, VEEV and WEEV). Multiple vaccine platforms have demonstrated promising pre-clinical and early clinical results. Although traditional live-attenuated and inactivated vaccines are available for at-risk personnel, limitations in terms of immunogenicity, reactogenicity and durability indicate the need for the development of next-generation vaccines. The most clinically advanced of these are MVA- and VLP-based vaccines, which have favourable safety profiles and immunogenicity in clinical trials. DNA and RNA vaccines offer potential advantages in terms of manufacturing, stability and deployment, particularly in settings with limited resources, but these require further clinical validation.

A recurring challenge across multiple vaccine platforms is developing multivalent vaccines capable of inducing balanced and durable immunogenicity against EEEV, VEEV and WEEV simultaneously. Several studies have reported evidence of immune interference resulting in lower immune responses compared to monovalent vaccines.

Overall, the development of future vaccines against encephalitic alphaviruses will require a balance of immunogenicity, safety, scalability and durability, while also establishing immune correlates of protection that can support regulatory approval and deployment.

## 8. Conclusions

EEEV, VEEV and WEEV are important emerging and re-emerging pathogens that can cause severe neurological disease in humans and animals. Although outbreaks are sporadic, the high mortality associated with EEEV, VEEV’s epidemic potential, and WEEV’s recent resurgence highlight their ongoing public health significance.

Despite significant advances in our understanding of their epidemiology, pathogenesis and immunology, major challenges remain. The lack of clearly defined immune correlates of protection continues to hinder vaccine development and evaluation, while the rarity and unpredictability of outbreaks make conducting conventional efficacy trials difficult. Diagnostic limitations and under-recognition of the disease burden further complicate surveillance and public health responses.

However, recent advances in vaccine development are encouraging, with a diverse pipeline of promising candidates emerging, including VLP, MVA, DNA, and rationally attenuated platforms. Progress is also being made in monoclonal antibody therapies, antiviral drug discovery, and improved diagnostic approaches.

Future efforts should focus on identifying immune correlates of protection, improving diagnostic capability and establishing regulatory pathways, such as the FDA Animal Rule, to facilitate vaccine licensure. Continued investment in surveillance, vaccine development and therapeutic research is essential in order to address the ongoing threat posed by encephalitic alphaviruses, particularly given the ongoing influence of climate change and ecological disruption on vector distribution and outbreak risk.

## Data Availability

No new data were created or analysed in this study. Data sharing is not applicable to this article.

## References

[1] Meyer K.F., Haring C.M., Howitt B. (1931). The etiology of epizootic encephalomyelitis of horses in the San Joaquin Valley, 1930. Science.

[2] Broeck G.T., Merrill M.H. (1933). A serological difference between Eastern and Western equine encephalomyelitis virus. Proc. Soc. Exp. Biol. Med..

[3] Kubes V., Ríos F.A. (1939). The causative agent of infectious equine encephalomyelitis in Venezuela. Science.

[4] Lennette E.H., Koprowski H. (1945). Serologic distinctness of Eastern, Western, and Venezuelan equine encephalomyelitis viruses. Proc. Soc. Exp. Biol. Med..

[5] Azar S.R., Campos R.K., Bergren N.A., Camargos V.N., Rossi S.L. (2020). Epidemic alphaviruses: Ecology, emergence and outbreaks. Microorganisms.

[6] Yang X., Ning Y., Xu C., Zhang Q., Dong Y., Wang Y., Zhang F., Lei Y., Ye W. (2026). Current status on encephalitic alphavirus vaccines development: Advances, challenges, and global health perspectives. Infect. Med..

[7] Strauss J.H., Strauss E.G. (1994). The alphaviruses: Gene expression, replication, and evolution. Microbiol. Mol. Biol. Rev..

[8] Aguilar P.V., Weaver S.C., Basler C.F. (2007). Capsid protein of Eastern equine encephalitis virus inhibits host cell gene expression. J. Virol..

[9] Atasheva S., Fish A., Fornerod M., Frolova E.I. (2010). Venezuelan equine encephalitis virus capsid protein forms a tetrameric complex with CRM1 and importin α/β that obstructs nuclear pore complex function. J. Virol..

[10] Garmashova N., Atasheva S., Kang W., Weaver S.C., Frolova E., Frolov I. (2007). Analysis of Venezuelan equine encephalitis virus capsid protein function in the inhibition of cellular transcription. J. Virol..

[11] Aguilar P.V., Leung L.W., Wang E., Weaver S.C., Basler C.F. (2008). A five-amino-acid deletion of the Eastern equine encephalitis virus capsid protein attenuates replication in mammalian systems but not in mosquito cells. J. Virol..

[12] Johnson B.J.B., Brubaker J.R., Roehrig J.T., Trent D.W. (1990). Variants of Venezuelan equine encephalitis virus that resist neutralization define a domain of the E2 glycoprotein. Virology.

[13] Meshram C.D., Shiliaev N., Frolova E.I., Frolov I. (2020). Hypervariable domain of nsP3 of Eastern equine encephalitis virus is a critical determinant of viral virulence. J. Virol..

[14] Ronca S.E., Dineley K.T., Paessler S. (2016). Neurological sequelae resulting from encephalitic alphavirus infection. Front. Microbiol..

[15] Stromberg Z.R., Fischer W., Bradfute S.B., Kubicek-Sutherland J.Z., Hraber P. (2020). Vaccine advances against Venezuelan, Eastern, and Western equine encephalitis viruses. Vaccines.

[16] Berge T.O., Banks I.S., Tigertt W.D. (1961). Attenuation of Venezuelan equine encephalomyelitis virus by in vitro cultivation in guinea-pig heart cells. Am. J. Epidemiol..

[17] McKinney R.W., Berge T.O., Sawyer W.D., Tigertt W.D., Crozier D. (1963). Use of an attenuated strain of Venezuelan equine encephalomyelitis virus for immunization in man. Am. J. Trop. Med. Hyg..

[18] Leparc-Goffart I., Nougairede A., Cassadou S., Prat C., de Lamballerie X. (2014). Chikungunya in the Americas. Lancet.

[19] Guzmán-Terán C., Calderón-Rangel A., Rodriguez-Morales A., Mattar S. (2020). Venezuelan equine encephalitis virus: The problem is not over for tropical America. Ann. Clin. Microbiol. Antimicrob..

[20] Aguilar P.V., Estrada-Franco J.G., Navarro-Lopez R., Ferro C., Haddow A.D., Weaver S.C. (2011). Endemic Venezuelan equine encephalitis in the Americas: Hidden under the dengue umbrella. Future Virol..

[21] Kumar B., Manuja A., Gulati B.R., Virmani N., Tripathi B.N. (2018). Zoonotic viral diseases of equines and their impact on human and animal health. Open Virol. J..

[22] Rusnak J.M., Glass P.J., Weaver S.C., Sabourin C.L., Glenn A.M., Klimstra W., Badorrek C.S., Nasar F., Ward L.A. (2019). Approach to strain selection and the propagation of viral stocks for Venezuelan equine encephalitis virus vaccine efficacy testing under the Animal Rule. Viruses.

[23] Silva M.L.C.R., Auguste A.J., Terzian A.C.B., Vedovello D., Riet-Correa F., Macário V.M.K., Mourão M.P.G., Ullmann L.S., Araújo J.P., Weaver S.C. (2017). Isolation and characterization of Madariaga virus from a horse in Paraíba state, Brazil. Transbound. Emerg. Dis..

[24] Zacks M.A., Paessler S. (2010). Encephalitic alphaviruses. Vet. Microbiol..

[25] Hahn C.S., Lustig S., Strauss E.G., Strauss J.H. (1988). Western equine encephalitis virus is a recombinant virus. Proc. Natl. Acad. Sci. USA.

[26] Weaver S.C., Kang W., Shirako Y., Rümenapf T., Strauss E.G., Strauss J.H. (1997). Recombinational history and molecular evolution of Western equine encephalomyelitis complex alphaviruses. J. Virol..

[27] Nagata L.P., Hu W.-G., Parker M., Chau D., Rayner G.A., Schmaltz F.L., Wong J.P. (2006). Infectivity variation and genetic diversity among strains of Western equine encephalitis virus. J. General Virol..

[28] Logue C.H., Bosio C.F., Welte T., Keene K.M., Ledermann J.P., Phillips A., Sheahan B.J., Pierro D.J., Marlenee N., Brault A.C. (2009). Virulence variation among isolates of Western equine encephalitis virus in an outbred mouse model. J. General Virol..

[29] Forrester N.L., Kenney J.L., Deardorff E., Wang E., Weaver S.C. (2008). Western equine encephalitis submergence: Lack of evidence for a decline in virus virulence. Virology.

[30] Bergren N.A., Haller S., Rossi S.L., Seymour R.L., Huang J., Miller A.L., Bowen R.A., Hartman D.A., Brault A.C., Weaver S.C. (2020). “Submergence” of Western equine encephalitis virus: Evidence of positive selection argues against genetic drift and fitness reductions. PLoS Pathog..

[31] Campos A.S., Franco A.C., Godinho F.M., Huff R., Candido D.S., da Cruz Cardoso J., Hua X., Claro I.M., Morais P., Franceschina C. (2024). Molecular epidemiology of Western equine encephalitis virus, South America, 2023–2024. Emerg. Infect. Dis..

[32] Thompson N.N., Auguste A.J., Travassos da Rosa A.P.A., Carrington C.V.F., Blitvich B.J., Chadee D.D., Tesh R.B., Weaver S.C., Adesiyun A.A. (2015). Seroepidemiology of selected alphaviruses and flaviviruses in bats in Trinidad. Zoonoses Public Health.

[33] Sotomayor-Bonilla J., Abella-Medrano C.A., Chaves A., Álvarez-Mendizábal P., Rico-Chávez Ó., Ibáñez-Bernal S., Rostal M.K., Ojeda-Flores R., Barbachano-Guerrero A., Gutiérrez-Espeleta G. (2017). Potential sympatric vectors and mammalian hosts of Venezuelan equine encephalitis virus in Southern Mexico. J. Wildl. Dis..

[34] Aréchiga-Ceballos N., Aguilar-Setién A. (2015). Alphaviral equine encephalomyelitis (Eastern, Western and Venezuelan). Rev. Sci. Tech.-Off. Int. Des. Epizoot..

[35] Carrara A.-S., Gonzales G., Ferro C., Tamayo M., Aronson J., Paessler S., Anishchenko M., Boshell J., Weaver S.C. (2005). Venezuelan equine encephalitis virus infection of spiny rats. Emerg. Infect. Dis..

[36] Deardorff E.R., Forrester N.L., Travassos da Rosa A.P.A., Estrada-Franco J.G., Navarro-Lopez R., Tesh R.B., Vasilakis N. (2009). Experimental infection of potential reservoir hosts with Venezuelan equine encephalitis virus, Mexico. Emerg. Infect. Dis..

[37] Coffey L.L., Carrara A.-S., Paessler S., Haynie M.L., Bradley R.D., Tesh R.B., Vasilakis N. (2004). Experimental Everglades virus infection of cotton rats (*Sigmodon hispidus*). Emerg. Infect. Dis..

[38] Ludlow M., Kortekaas J., Herden C., Hoffmann B., Tappe D., Trebst C., Griffin D.E., Brindle H.E., Solomon T., Brown A.S. (2016). Neurotropic virus infections as the cause of immediate and delayed neuropathology. Acta Neuropathol..

[39] Arrigo N.C., Adams A.P., Watts D.M., Newman P.C., Vasilakis N. (2010). Cotton rats and house sparrows as hosts for North and South American strains of Eastern equine encephalitis virus. Emerg. Infect. Dis..

[40] Mitchell C.J., McLean R.G., Nasci R.S., Crans W.J., Smith G.C., Caccamise D.F. (1993). Susceptibility parameters of Aedes albopictus to peroral infection with Eastern equine encephalitis virus. J. Med. Entomol..

[41] Woodson C.M., Carney S.K., Kehn-Hall K. (2025). Neuropathogenesis of encephalitic alphaviruses in non-human primate and mouse models of infection. Pathogens.

[42] Avilés G., Sabattini M.S., Mitchell C.J. (1992). Transmission of Western equine encephalomyelitis virus by Argentine Aedes albifasciatus (Diptera: Culicidae). J. Med. Entomol..

[43] Reyna R.A., Weaver S.C. (2023). Sequelae and animal modeling of encephalitic alphavirus infections. Viruses.

[44] Lucas C.J., Morrison T.E. (2022). Chapter two—Animal models of alphavirus infection and human disease. Adv. Virus Res..

[45] Phelps A.L., O’Brien L.M., Eastaugh L.S., Davies C., Lever M.S., Ennis J., Zeitlin L., Nunez A., Ulaeto D.O. (2019). Aerosol infection of Balb/c mice with Eastern equine encephalitis virus; susceptibility and lethality. Virol. J..

[46] London W.T., Levitt N.H., Kent S.G., Wong V.G., Sever J.L. (1977). Congenital cerebral and ocular malformations induced in rhesus monkeys by Venezuelan equine encephalitis virus. Teratology.

[47] Moreland A.F., Schimpff R.D., Gaskin J.M. (1979). Fetal mortality and malformations associated with experimental infections of Western equine encephalomyelitis vaccine virus in rhesus monkeys (*Macaca mulatta*). Teratology.

[48] Elliott K.C., Saunders D., Mattapallil J.J. (2026). Venezuelan equine encephalitis virus: Novel live-attenuated vaccines for inducing complete protective immunity. npj Viruses.

[49] Forshey B.M., Guevara C., Laguna-Torres V.A., Cespedes M., Vargas J., Gianella A., Vallejo E., Madrid C., Aguayo N., Gotuzzo E. (2010). Arboviral etiologies of acute febrile illnesses in Western South America, 2000-2007. PLoS Neglected Trop. Dis..

[50] Carrera J.-P., Pittí Y., Molares-Martínez J.C., Casal E., Pereyra-Elias R., Saenz L., Guerrero I., Galué J., Rodriguez-Alvarez F., Jackman C. (2020). Clinical and serological findings of Madariaga and Venezuelan equine encephalitis viral infections: A follow-up study 5 years after an outbreak in Panama. Open Forum Infect. Dis..

[51] Wilson A.L., Courtenay O., Kelly-Hope L.A., Scott T.W., Takken W., Torr S.J., Lindsay S.W. (2020). The importance of vector control for the control and elimination of vector-borne diseases. PLoS Neglected Trop. Dis..

[52] CDC Historic Data (2003–2024). https://www.cdc.gov/eastern-equine-encephalitis/data-maps/historic-data.html.

[53] Carrera J.-P., Forrester N., Wang E., Vittor A.Y., Haddow A.D., López-Vergès S., Abadía I., Castaño E., Sosa N., Báez C. (2013). Eastern equine encephalitis in Latin America. N. Engl. J. Med..

[54] Carrera J.-P., Araúz D., Rojas A., Cardozo F., Stittleburg V., Morales Claro I., Galue J., Lezcano-Coba C., Romero Rebello Moreira F., Felipe-Rivera L. (2023). Real-time RT-PCR for Venezuelan equine encephalitis complex, Madariaga, and Eastern equine encephalitis viruses: Application in human and mosquito public health surveillance in Panama. J. Clin. Microbiol..

[55] Bergren N.A., Auguste A.J., Forrester N.L., Negi S.S., Braun W.A., Weaver S.C. (2014). Western equine encephalitis virus: Evolutionary analysis of a declining alphavirus based on complete genome sequences. J. Virol..

[56] Eberly A.R. (2024). Medically important alphaviruses in the United States and how to test for them. Clin. Microbiol. Newsl..

[57] Salimi H., Cain M.D., Klein R.S. (2016). Encephalitic arboviruses: Emergence, clinical presentation, and neuropathogenesis. Neurotherapeutics.

[58] Weaver S.C., Tesh R.B., Shope R.E. (2006). Chapter 74—Alphavirus infections. Trop. Infect. Dis. (Second Ed.) Princ. Pathog. Pract..

[59] VanderGiessen M., de Jager C., Leighton J., Xie H., Theus M., Johnson E., Kehn-Hall K. (2024). Neurological manifestations of encephalitic alphaviruses, traumatic brain injuries, and organophosphorus nerve agent exposure. Front. Neurosci..

[60] Deresiewicz R.L., Thaler S.J., Hsu L., Zamani A.A. (1997). Clinical and neuroradiographic manifestations of eastern equine encephalitis. N. Engl. J. Med..

[61] Bantle C.M., Phillips A.T., Smeyne R.J., Rocha S.M., Olson K.E., Tjalkens R.B. (2019). Infection with mosquito-borne alphavirus induces selective loss of dopaminergic neurons, neuroinflammation and widespread protein aggregation. npj Park. Dis..

[62] Bantle C.M., Rocha S.M., French C.T., Phillips A.T., Tran K., Olson K.E., Bass T.A., Aboellail T., Smeyne R.J., Tjalkens R.B. (2021). Astrocyte inflammatory signaling mediates α-synuclein aggregation and dopaminergic neuronal loss following viral encephalitis. Exp. Neurol..

[63] Charles P.C., Trgovcich J., Davis N.L., Johnston R.E. (2001). Immunopathogenesis and immune modulation of Venezuelan equine encephalitis virus-induced disease in the mouse. Virology.

[64] Yun N.E., Peng B.-H., Bertke A.S., Borisevich V., Smith J.K., Smith J.N., Poussard A.L., Salazar M., Judy B.M., Zacks M.A. (2009). CD4+ T cells provide protection against acute lethal encephalitis caused by Venezuelan equine encephalitis virus. Vaccine.

[65] Taylor K., Kolokoltsova O., Ronca S.E., Estes M., Paessler S. (2017). Live, attenuated Venezuelan equine encephalitis virus vaccine (TC83) causes persistent brain infection in mice with non-functional αβ T-cells. Front. Microbiol..

[66] Gardner C.L., Ebel G.D., Ryman K.D., Klimstra W.B. (2011). Heparan sulfate binding by natural Eastern equine encephalitis viruses promotes neurovirulence. Proc. Natl. Acad. Sci. USA.

[67] Salimi H., Cain M.D., Jiang X., Roth R.A., Beatty W.L., Sun C., Klimstra W.B., Hou J., Klein R.S. (2020). Encephalitic alphaviruses exploit caveola-mediated transcytosis at the blood-brain barrier for central nervous system entry. mBio.

[68] Phillips A.T., Rico A.B., Stauft C.B., Hammond S.L., Aboellail T.A., Tjalkens R.B., Olson K.E. (2016). Entry sites of Venezuelan and Western equine encephalitis viruses in the mouse central nervous system following peripheral infection. J. Virol..

[69] Bhalla N., Sun C., Lam L.K.M., Gardner C.L., Ryman K.D., Klimstra W.B. (2016). Host translation shutoff mediated by non-structural protein 2 is a critical factor in the antiviral state resistance of Venezuelan equine encephalitis virus. Virology.

[70] Parashar B., Malviya R., Sridhar S.B., Wadhwa T., Talath S., Shareef J. (2025). Eastern equine encephalitis virus: Pathogenesis, immune response, and clinical manifestations. Infect. Med..

[71] Yin J., Gardner C.L., Burke C.W., Ryman K.D., Klimstra W.B. (2009). Similarities and differences in antagonism of neuron alpha/beta interferon responses by Venezuelan equine encephalitis and Sindbis alphaviruses. J. Virol..

[72] Trobaugh D., Sun C., Klimstra W. (2018). Eastern equine encephalitis virus evades induction of the host immune response through miR-142-3p restriction of myeloid cell replication. J. Immunol..

[73] Zubair A.S., McAlpine L.S., Gobeske K.T. (2024). Virology, ecology, epidemiology, pathology, and treatment of eastern equine encephalitis. J. Neurol. Sci..

[74] Griffin D.E., Levine B., Tyor W.R., Tucker P.C., Hardwick J.M. (1994). Age-dependent susceptibility to fatal encephalitis: Alphavirus infection of neurons. Arch. Virol. Suppl..

[75] Griffin D.E. (1995). Roles and reactivities of antibodies to alphaviruses. Semin. Virol..

[76] Steele K.E., Twenhafel N.A. (2010). Review paper: Pathology of animal models of alphavirus encephalitis. Vet. Pathol..

[77] Ronca S.E., Smith J., Koma T., Miller M.M., Yun N., Dineley K.T., Paessler S. (2017). Mouse model of neurological complications resulting from encephalitic alphavirus infection. Front. Microbiol..

[78] Labrada L., Liang X.H., Zheng W., Johnston C., Levine B. (2002). Age-dependent resistance to lethal alphavirus encephalitis in mice: Analysis of gene expression in the central nervous system and identification of a novel interferon-inducible protective gene, mouse ISG12. J. Virol..

[79] Vogel P., Kell W.M., Fritz D.L., Parker M.D., Schoepp R.J. (2005). Early events in the pathogenesis of Eastern equine encephalitis virus in mice. Am. J. Pathol..

[80] Roy C.J., Reed D.S., Wilhelmsen C.L., Hartings J., Norris S., Steele K.E. (2009). Pathogenesis of aerosolized Eastern equine encephalitis virus infection in guinea pigs. Virol. J..

[81] Albe J.R., Ma H., Gilliland T.H., McMillen C.M., Gardner C.L., Boyles D.A., Cottie E.L., Dunn M.D., Lundy J.D., O’Malley K.J. (2021). Physiological and immunological changes in the brain associated with lethal Eastern equine encephalitis virus in macaques. PLoS Pathog..

[82] Smith D.R., Schmaljohn C.S., Badger C., Ostrowski K., Zeng X., Grimes S.D., Rayner J.O. (2020). Comparative pathology study of Venezuelan, Eastern, and Western equine encephalitis viruses in non-human primates. Antivir. Res..

[83] Gardner C.L., Sun C., Dunn M.D., Gilliland T.C., Trobaugh D.W., Terada Y., Reed D.S., Hartman A.L., Klimstra W.B. (2022). In vitro and in vivo phenotypes of Venezuelan, Eastern and Western equine encephalitis viruses derived from cDNA clones of human isolates. Viruses.

[84] Sánchez-Seco M.P., Rosario D., Quiroz E., Guzmán G., Tenorio A. (2001). A generic nested-RT-PCR followed by sequencing for detection and identification of members of the alphavirus genus. J. Virol. Methods.

[85] Eshoo M.W., Whitehouse C.A., Zoll S.T., Massire C., Pennella T.-T.D., Blyn L.B., Sampath R., Hall T.A., Ecker J.A., Desai A. (2007). Direct broad-range detection of alphaviruses in mosquito extracts. Virology.

[86] Ni H., Yun N.E., Zacks M.A., Weaver S.C., Tesh R.B., da Rosa A.P.T., Powers A.M., Frolov I., Paessler S. (2007). Recombinant alphaviruses are safe and useful serological diagnostic tools. Am. J. Trop. Med. Hyg..

[87] Lambert A.J., Martin D.A., Lanciotti R.S. (2003). Detection of North American Eastern and Western equine encephalitis viruses by nucleic acid amplification assays. J. Clin. Microbiol..

[88] Linssen B., Kinney R.M., Aguilar P., Russell K.L., Watts D.M., Kaaden O.-R., Pfeffer M. (2000). Development of reverse transcription-PCR assays specific for detection of equine encephalitis viruses. J. Clin. Microbiol..

[89] Williamson L.E., Reeder K.M., Bailey K., Tran M.H., Roy V., Fouch M.E., Kose N., Trivette A., Nargi R.S., Winkler E.S. (2021). Therapeutic alphavirus cross-reactive E1 human antibodies inhibit viral egress. Cell.

[90] Fox J.M., Long F., Edeling M.A., Lin H., van Duijl-Richter M.K.S., Fong R.H., Kahle K.M., Smit J.M., Jin J., Simmons G. (2015). Broadly neutralizing alphavirus antibodies bind an epitope on E2 and inhibit entry and egress. Cell.

[91] Fischer C., Bozza F., Merino Merino X.J., Pedroso C., de Oliveira Filho E.F., Moreira-Soto A., Schwalb A., de Lamballerie X., Netto E.M., Bozza P.T. (2020). Robustness of serologic investigations for Chikungunya and Mayaro viruses following coemergence. mSphere.

[92] Sardesai A.M., Brown N.M., Menon D.K. (2002). Deliberate release of biological agents. Anaesthesia.

[93] CDC Treatment and Prevention of Eastern Equine Encephalitis. https://www.cdc.gov/eastern-equine-encephalitis/hcp/treatment-prevention/index.html.

[94] Coates E.E., Edupuganti S., Chen G.L., Happe M., Strom L., Widge A., Florez M.B., Cox J.H., Gordon I., Plummer S. (2022). Safety and immunogenicity of a trivalent virus-like particle vaccine against Western, Eastern, and Venezuelan equine encephalitis viruses: A phase 1, open-label, dose-escalation, randomised clinical trial. Lancet Infect. Dis..

[95] Kafai N.M., Williamson L.E., Binshtein E., Sukupolvi-Petty S., Gardner C.L., Liu J., Mackin S., Kim A.S., Kose N., Carnahan R.H. (2022). Neutralizing antibodies protect mice against Venezuelan equine encephalitis virus aerosol challenge. J. Exp. Med..

[96] Verwimp S., Wagoner J., Arenas E.G., De Coninck L., Abdelnabi R., Hyde J.L., Schiffer J.T., White J.M., Matthijnssens J., Neyts J. (2025). Combinations of approved oral nucleoside analogues confer potent suppression of alphaviruses in vitro and in vivo. Antivir. Res..

[97] Pareek A., Kumar R., Mudgal R., Neetu N., Sharma M., Kumar P., Tomar S. (2022). Alphavirus antivirals targeting RNA-dependent RNA polymerase domain of nsP4 divulged using surface plasmon resonance. FEBS J..

[98] Hong J.P., McCarthy M.K., Davenport B.J., Morrison T.E., Diamond M.S. (2019). Clearance of Chikungunya virus infection in lymphoid tissues is promoted by treatment with an agonistic anti-CD137 antibody. J. Virol..

[99] Nagata L.P., Wong J.P., Hu W.-G., Wu J.Q. (2013). Vaccines and therapeutics for the encephalitic alphaviruses. Future Virol..

[100] Mussgay M., Suárez O. (1962). Studies with a pathogenic and an attenuated strain of Venezuelan equine encephalitis virus and *Aedes aegypti* (L.) mosquitoes. Arch. Für Die Gesamte Virusforsch..

[101] Edelman R., Ascher M.S., Oster C.N., Ramsburg H.H., Cole F.E., Eddy G.A. (1979). Evaluation in humans of a new, inactivated vaccine for Venezuelan equine encephalitis virus (C-84). J. Infect. Dis..

[102] Pittman P.R., Makuch R.S., Mangiafico J.A., Cannon T.L., Gibbs P.H., Peters C.J. (1996). Long-term duration of detectable neutralizing antibodies after administration of live-attenuated VEE vaccine and following booster vaccination with inactivated VEE vaccine. Vaccine.

[103] Jahrling P.B., Stephenson E.H. (1984). Protective efficacies of live attenuated and formaldehyde-inactivated Venezuelan equine encephalitis virus vaccines against aerosol challenge in hamsters. J. Clin. Microbiol..

[104] Kinney R.M., Tsuchiya K.R., Sneider J.M., Trent D.W. (1992). Molecular evidence for the origin of the widespread Venezuelan equine encephalitis epizootic of 1969 to 1972. J. General Virol..

[105] Davis N.L., Brown K.W., Greenwald G.F., Zajac A.J., Zacny V.L., Smith J.F., Johnston R.E. (1995). Attenuated mutants of Venezuelan equine encephalitis virus containing lethal mutations in the PE2 cleavage signal combined with a second-site suppressor mutation in E1. Virology.

[106] Fine D.L., Roberts B.A., Teehee M.L., Terpening S.J., Kelly C.L.H., Raetz J.L., Baker D.C., Powers A.M., Bowen R.A. (2007). Venezuelan equine encephalitis virus vaccine candidate (V3526) safety, immunogenicity and efficacy in horses. Vaccine.

[107] Martin S.S., Bakken R.R., Lind C.M., Reed D.S., Price J.L., Koeller C.A., Parker M.D., Hart M.K., Fine D.L. (2009). Telemetric analysis to detect febrile responses in mice following vaccination with a live-attenuated virus vaccine. Vaccine.

[108] Sharma A., Gupta P., Glass P.J., Parker M.D., Maheshwari R.K. (2011). Safety and protective efficacy of INA-inactivated Venezuelan equine encephalitis virus: Implication in vaccine development. Vaccine.

[109] Gupta P., Sharma A., Spurgers K.B., Bakken R.R., Eccleston L.T., Cohen J.W., Honnold S.P., Glass P.J., Maheshwari R.K. (2016). 1,5-iodonaphthyl azide-inactivated V3526 protects against aerosol challenge with virulent Venezuelan equine encephalitis virus. Vaccine.

[110] Haines C.A., Campos R.K., Azar S.R., Warmbrod K.L., Kautz T.F., Forrester N.L., Rossi S.L. (2022). Venezuelan equine encephalitis virus V3526 vaccine RNA-dependent RNA polymerase mutants increase vaccine safety through restricted tissue tropism in a murine model. Zoonoses.

[111] Dupuy L.C., Richards M.J., Ellefsen B., Chau L., Luxembourg A., Hannaman D., Livingston B.D., Schmaljohn C.S. (2011). A DNA vaccine for Venezuelan equine encephalitis virus delivered by intramuscular electroporation elicits high levels of neutralizing antibodies in multiple animal models and provides protective immunity to mice and nonhuman primates. Clin. Vaccine Immunol..

[112] Dupuy L.C., Richards M.J., Livingston B.D., Hannaman D., Schmaljohn C.S. (2018). A multiagent alphavirus DNA vaccine delivered by intramuscular electroporation elicits robust and durable virus-specific immune responses in mice and rabbits and completely protects mice against lethal Venezuelan, Western, and Eastern equine encephalitis virus aerosol challenges. J. Immunol. Res..

[113] Hannaman D., Dupuy L.C., Ellefsen B., Schmaljohn C.S. (2016). A phase 1 clinical trial of a DNA vaccine for Venezuelan equine encephalitis delivered by intramuscular or intradermal electroporation. Vaccine.

[114] Suschak J.J., Bixler S.L., Badger C.V., Spik K.W., Kwilas S.A., Rossi F.D., Twenhafel N., Adams M.L., Shoemaker C.J., Spiegel E. (2022). A DNA vaccine targeting VEE virus delivered by needle-free jet-injection protects macaques against aerosol challenge. npj Vaccines.

[115] Tretyakova I., Lukashevich I.S., Glass P., Wang E., Weaver S., Pushko P. (2013). Novel vaccine against Venezuelan equine encephalitis combines advantages of DNA immunization and a live attenuated vaccine. Vaccine.

[116] Rouxel R.N., Tafalla C., Mérour E., Leal E., Biacchesi S., Brémont M. (2016). Attenuated infectious hematopoietic necrosis virus with rearranged gene order as potential vaccine. J. Virol..

[117] Flanagan E.B., Schoeb T.R., Wertz G.W. (2003). Vesicular stomatitis viruses with rearranged genomes have altered invasiveness and neuropathogenesis in mice. J. Virol..

[118] Tretyakova I., Tibbens A., Jokinen J.D., Johnson D.M., Lukashevich I.S., Pushko P. (2019). Novel DNA-launched Venezuelan equine encephalitis virus vaccine with rearranged genome. Vaccine.

[119] Johnson D.M., Sokoloski K.J., Jokinen J.D., Pfeffer T.L., Chu Y.-K., Adcock R.S., Chung D., Tretyakova I., Pushko P., Lukashevich I.S. (2020). Advanced safety and genetic stability in mice of a novel DNA-launched Venezuelan equine encephalitis virus vaccine with rearranged structural genes. Vaccines.

[120] Tretyakova I., Plante K.S., Rossi S.L., Lawrence W.S., Peel J.E., Gudjohnsen S., Wang E., Mirchandani D., Tibbens A., Lamichhane T.N. (2020). Venezuelan equine encephalitis vaccine with rearranged genome resists reversion and protects non-human primates from viremia after aerosol challenge. Vaccine.

[121] Tretyakova I., Tomai M., Vasilakos J., Pushko P. (2022). Live-attenuated VEEV vaccine delivered by iDNA using microneedles is immunogenic in rabbits. Front. Trop. Dis..

[122] Henning L., Endt K., Steigerwald R., Anderson M., Volkmann A. (2020). A monovalent and trivalent MVA-based vaccine completely protects mice against lethal Venezuelan, Western, and Eastern equine encephalitis virus aerosol challenge. Front. Immunol..

[123] Fierro C., Weidenthaler H., Vidojkovic S., Schmidt D., Gafoor Z., Stroukova D., Zwiers S., Müller J., Volkmann A. (2024). Safety and immunogenicity of a novel trivalent recombinant MVA-based equine encephalitis virus vaccine: A phase 1 clinical trial. Vaccine.

[124] Ko S.-Y., Akahata W., Yang E.S., Kong W.-P., Burke C.W., Honnold S.P., Nichols D.K., Huang Y.-J.S., Schieber G.L., Carlton K. (2019). A virus-like particle vaccine prevents equine encephalitis virus infection in nonhuman primates. Sci. Transl. Med..

[125] Bennett S.R., McCarty J.M., Ramanathan R., Mendy J., Richardson J.S., Smith J., Alexander J., Ledgerwood J.E., de Lame P.-A., Tredo S.R. (2022). Safety and immunogenicity of PXVX0317, an aluminium hydroxide-adjuvanted Chikungunya virus-like particle vaccine: A randomised, double-blind, parallel-group, phase 2 trial. Lancet Infect. Dis..

[126] Reisler R.B., Gibbs P.H., Danner D.K., Boudreau E.F. (2012). Immune interference in the setting of same-day administration of two similar inactivated alphavirus vaccines: Eastern equine and western equine encephalitis. Vaccine.

[127] Reed D.S., Glass P.J., Bakken R.R., Barth J.F., Lind C.M., da Silva L., Hart M.K., Rayner J., Alterson K., Custer M. (2014). Combined alphavirus replicon particle vaccine induces durable and cross-protective immune responses against equine encephalitis viruses. J. Virol..

[128] Burke C.W., Erwin-Cohen R.A., Goodson A.I., Wilhelmsen C., Edmundson J.A., White C.E., Glass P.J. (2022). Efficacy of Western, Eastern, and Venezuelan equine encephalitis (WEVEE) virus-replicon particle (VRP) vaccine against WEEV in a non-human primate animal model. Viruses.

[129] Robinson D.M., Berman S., Lowenthal J.P., Hetrick F.M. (1966). Western equine encephalomyelitis vaccine produced in chick embryo cell cultures. Appl. Environ. Microbiol..

[130] Hu W.-G., Steigerwald R., Kalla M., Volkmann A., Noll D., Nagata L.P. (2018). Protective efficacy of monovalent and trivalent recombinant MVA-based vaccines against three encephalitic alphaviruses. Vaccine.

[131] Keshtkar-Jahromi M., Reisler R.B., Haller J.M., Clizbe D.P., Rivard R.G., Cardile A.P., Pierson B.C., Norris S., Saunders D., Pittman P.R. (2020). The western equine encephalitis lyophilized, inactivated vaccine: An update on safety and immunogenicity. Front. Immunol..

[132] Beddingfield B.J., Plante K.S., Plante J.A., Weaver S.C., Bose S., Krzykwa C., Chirichella N., Redmann R.K., Seiler S.Z., Dufour J. (2024). MVA-based vaccines are protective against lethal Eastern equine encephalitis virus aerosol challenge in cynomolgus macaques. npj Vaccines.

[133] Trobaugh D.W., Sun C., Dunn M.D., Reed D.S., Klimstra W.B. (2019). Rational design of a live-attenuated Eastern equine encephalitis virus vaccine through informed mutation of virulence determinants. PLoS Pathog..

[134] Pandya J., Gorchakov R., Wang E., Leal G., Weaver S.C. (2012). A vaccine candidate for Eastern equine encephalitis virus based on IRES-mediated attenuation. Vaccine.

[135] Honnold S.P., Bakken R.R., Fisher D., Lind C.M., Cohen J.W., Eccleston L.T., Spurgers K.B., Maheshwari R.K., Glass P.J. (2014). Second generation inactivated Eastern equine encephalitis virus vaccine candidates protect mice against a lethal aerosol challenge. PLoS ONE.

[136] Wang E., Petrakova O., Adams A.P., Aguilar P.V., Kang W., Paessler S., Volk S.M., Frolov I., Weaver S.C. (2007). Chimeric Sindbis/Eastern equine encephalitis vaccine candidates are highly attenuated and immunogenic in mice. Vaccine.

[137] Roy C.J., Adams A.P., Wang E., Leal G., Seymour R.L., Sivasubramani S.K., Mega W., Frolov I., Didier P.J., Weaver S.C. (2013). A chimeric Sindbis-based vaccine protects cynomolgus macaques against a lethal aerosol challenge of Eastern equine encephalitis virus. Vaccine.

[138] Erasmus J.H., Seymour R.L., Kaelber J.T., Kim D.Y., Leal G., Sherman M.B., Frolov I., Chiu W., Weaver S.C., Nasar F. (2018). Novel insect-specific Eilat virus-based chimeric vaccine candidates provide durable, mono- and multivalent, single-dose protection against lethal alphavirus challenge. J. Virol..

[139] Nasar F., Matassov D., Seymour R.L., Latham T., Gorchakov R.V., Nowak R.M., Leal G., Hamm S., Eldridge J.H., Tesh R.B. (2017). Recombinant Isfahan virus and vesicular stomatitis virus vaccine vectors provide durable, multivalent, single-dose protection against lethal alphavirus challenge. J. Virol..

[140] Phillips A.T., Schountz T., Toth A.M., Rico A.B., Jarvis D.L., Powers A.M., Olson K.E. (2014). Liposome-antigen-nucleic acid complexes protect mice from lethal challenge with Western and Eastern equine encephalitis viruses. J. Virol..

[141] Powers A.M. (2022). Resurgence of interest in Eastern equine encephalitis virus vaccine development. J. Med. Entomol..

[142] Hatami J., de Bruin A.C.M., Bánki Z., Rey F.A., Gerold G. (2026). Antibody cross-reactivity among alphaviruses in clinical practice. Trends Microbiol..

